# Post-endocytic sorting of Plexin-D1 controls signal transduction and development of axonal and vascular circuits

**DOI:** 10.1038/ncomms14508

**Published:** 2017-02-22

**Authors:** Katja Burk, Erik Mire, Anaïs Bellon, Mélanie Hocine, Jeremy Guillot, Filipa Moraes, Yutaka Yoshida, Michael Simons, Sophie Chauvet, Fanny Mann

**Affiliations:** 1 Aix Marseille Univ, CNRS, IBDM, Marseille 13288, France; 2 Yale Cardiovascular Research Center, Section of Cardiovascular Medicine, Department of Internal Medicine, Yale University School of Medicine, New Haven, Connecticut 06511, USA; 3 Division of Developmental Biology, Cincinnati Children's Hospital Medical Center, Cincinnati, Ohio 45229, USA; 4 Department of Cell Biology Yale University School of Medicine, New Haven, Connecticut 06511, USA

## Abstract

Local endocytic events involving receptors for axon guidance cues play a central role in controlling growth cone behaviour. Yet, little is known about the fate of internalized receptors, and whether the sorting events directing them to distinct endosomal pathways control guidance decisions. Here, we show that the receptor Plexin-D1 contains a sorting motif that interacts with the adaptor protein GIPC1 to facilitate transport to recycling endosomes. This sorting process promotes colocalization of Plexin-D1 with vesicular pools of active R-ras, leading to its inactivation. In the absence of interaction with GIPC1, missorting of Plexin-D1 results in loss of signalling activity. Consequently, *Gipc1* mutant mice show specific defects in axonal projections, as well as vascular structures, that rely on Plexin-D1 signalling for their development. Thus, intracellular sorting steps that occur after receptor internalization by endocytosis provide a critical level of control of cellular responses to guidance signals.

## Abstract (web summary)

Molecular mechanisms controlling axonal growth cone behaviour are only partially understood. Here the authors reveal a role of an adaptor protein GIPC1 in Plexin-D1 receptor recycling, and show that this process is required for axon track formation and vascular patterning in mice.

The nervous system wires itself with remarkable precision due to the homing behaviour of axonal growth cones, whose function is dependent on membrane trafficking events. Exocytosis and endocytosis are both essential to regulate growth cone morphology and adhesive properties during axon outgrowth and guidance^1^,^2^,^3^. In particular, during chemotactic guidance, spatial asymmetry in membrane trafficking across the growth cone drives its turning response to the side with increased exocytosis, or decreased endocytosis^4^,^5^.

In addition to acting as a driving force for axon development, membrane trafficking also regulates the dynamics of cell surface receptors for extracellular ligands^6^. Endocytosis of ligand–receptor complexes from the plasma membrane has been primarily associated with desensitization of axonal responses to axon guidance cues^7^. However, endocytosis also critically regulates signalling from guidance cue receptors. For example, the Frizzled3 receptor requires internalization from the cell surface to activate planar cell polarity signalling during Wnt-promoted growth of spinal commissural axons^8^, as does the Robo receptor to recruit Son of Sevenless, a downstream effector of repulsive Slit signalling at the midline^9^. Shortly following endocytosis, internalized receptors are delivered to early endosomes that constitute the primary sorting station along the post-endocytic pathway. Sorting events initiated at this compartment determine the fate of internalized receptors, destining them either for recycling to the plasma membrane, transport to the Golgi or degradation in lysosomes. Potentially, signalling activity can be regulated at the level of post-endocytic sorting through spatial relocation of receptors and interaction with signalling molecules that are compartmentalized into specific endosomal vesicles^10^. However, little is currently known about the fate of guidance cue receptors endocytosed at the growth cone and whether post-endocytic sorting events play a role in dictating their signalling responses.

The Semaphorins define a large family of guidance cues that can elicit growth cone collapse and repulsive turning. The prototypic semaphorin, Sema3A, induces internalization of its receptor complex during repulsive axon guidance^11^. A recent study reported that the two Sema3A co-receptors, Neuropilin-1 and L1CAM, segregate in endosomes of different lipid composition after their co-endocytosis in growth cones of embryonic sensory neurons^12^. Interestingly, the adhesion molecule TAG-1 (transient axonal glycoprotein-1), which is required for Sema3A-induced collapse of sensory growth cones, has been found to facilitate endocytosis of the Neuropilin-1/L1CAM complex and to mediate the subsequent segregation of the two proteins into different endosomal populations^12^,^13^. While this suggests a link between intracellular trafficking of co-receptor proteins and Semaphorin signalling, exactly how these two events are related to each other is unclear. Indeed, it remains to be determined how the signal-transducing elements of the Semaphorin receptor complexes, the Plexins, are trafficked inside the growth cone and whether endosomal sorting directly controls Plexin receptor activity and signal transduction.

Here, we focus on Plexin-D1, the cell surface receptor for the Semaphorin 3E (Sema3E) ligand, to investigate the interplay between post-endocytic sorting and signalling in growth cone guidance. Sema3E has the unique ability among class 3 semaphorins to bind directly to Plexin-D1 without requiring a Neuropilin as a co-receptor^14^. Sema3E-dependent activation of Plexin-D1 induces cell repulsion and is involved in various aspects of neuronal wiring, from axon growth and guidance to synapse formation^15^. Here we identify a sorting mechanism involving the PDZ domain-containing protein GIPC1 (also known as Synectin) that regulates transport of ligand-activated Plexin-D1 at trafficking checkpoints downstream of endocytosis. Interfering with this mechanism reveals that Plexin-D1 signalling in growth cones is initiated from endocytic recycling compartments and missorting of the internalized receptor causes loss of cell response to Sema3E and specific axon guidance errors *in vivo*. This GIPC1-dependent mechanism also regulates blood vessel guidance *in vivo*. Thus, we propose that the precise sorting of guidance cue receptors along the endosomal pathway provides an important level of regulation of the signalling pathways that governs the wiring of neuronal and vascular circuits.

## Results

### Sema3E-induced growth cone collapse requires endocytosis

**Figure 1:**
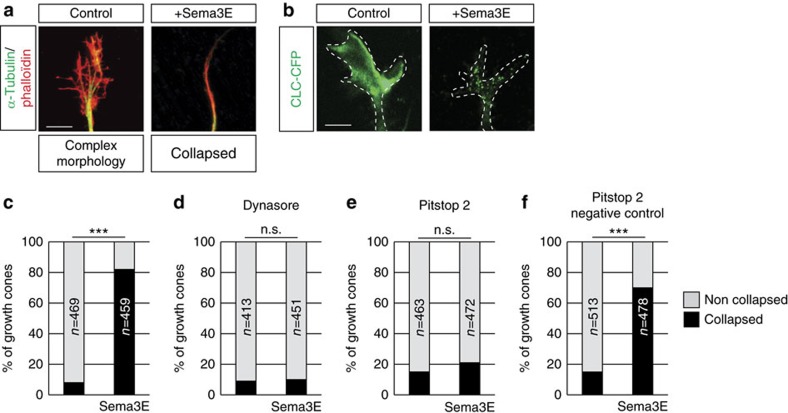
Endocytosis is required for Sema3E-induced growth cone collapse. (**a**) Collapse assay performed on E15.5 Pir neurons identified by tubulin in the presence or absence of Sema3E (20 min of treatment). Phalloidin staining shows the complex morphology of growth cones in the control condition and the collapsed morphology in the presence of Sema3E. (**b**) Image of growth cones of cultured E15.5 Pir neurons expressing clathrin light chain-CFP (CLC-CFP), with or without Sema3E (10 min of treatment). (**c**–**f**) Quantification of the percentage of collapsed growth cones in control cultures and in response to Sema3E (20 min of treatment). Sema3E-induced collapse was blocked by the endocytosis inhibitors dynasore and Pitstop 2; *n*=number of growth cones analysed per condition in three independent experiments. The χ^2^ test, ***P<0.0001. Scale bars, 10 μm. See also Supplementary Fig. 1.

Since previous studies involved endocytosis in regulating guidance receptor signalling, we sought to carefully characterize the role of endocytic trafficking in the repulsive response of growth cones to Sema3E. For this, 10 nM Sema3E was bath-applied to Plexin-D1-expressing neurons isolated from mouse embryonic day (E) 15.5 piriform cortex (Pir)^16^. After 10 min, the number of collapsed growth cones rose to 50%, and reached a maximum of ∼85% after 20 min (Fig. 1a and Supplementary Fig. 1). To examine receptor-mediated endocytosis, we expressed in neurons a clathrin light chain-cyan fluorescent protein (CLC-CFP) fusion protein. A 10-min treatment with Sema3E induced the redistribution of clathrin into a punctate fluorescent pattern revealing hot spots of endocytosis that were already visible in growth cones that had not yet collapsed (Fig. 1b). We next tested the functional requirement of endocytosis for Sema3E-induced growth cone collapse by using pharmacological inhibitors of clathrin (Pitstop 2 and a negative control)^17^ and dynamin (dynasore^18^). Blocking clathrin- and dynamin-dependent endocytosis completely suppressed the growth cone collapsing effect of Sema3E (Fig. 1c–f).

### Sema3E promotes endocytosis of Plexin-D1 in the growth cone

**Figure 2:**
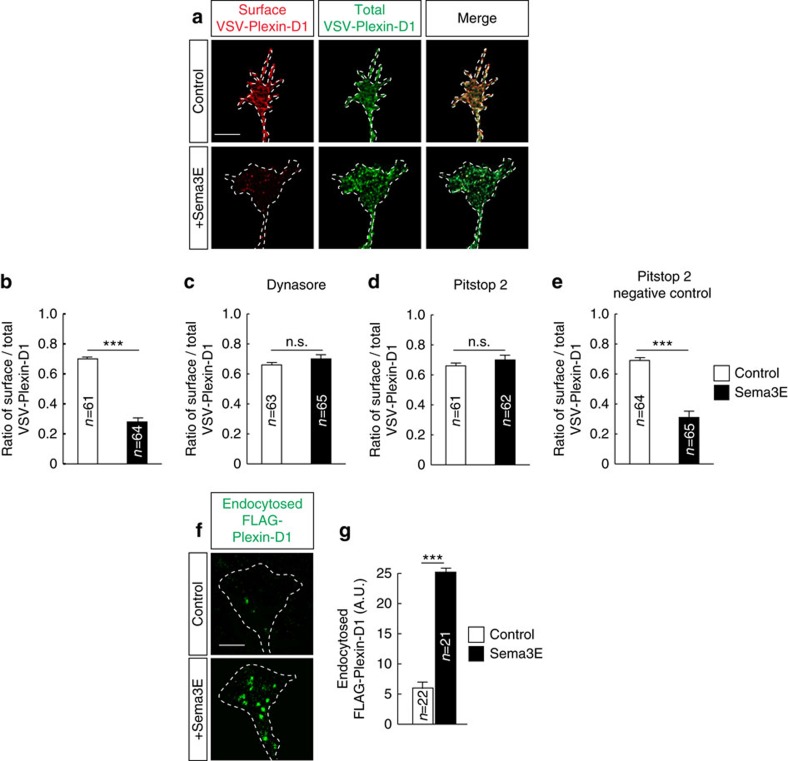
Sema3E induces Plexin-D1 endocytosis. (**a**) Examples of growth cones from E15.5 Pir neurons showing cell surface localization (Control) and internalization (+Sema3E) of VSV-Plexin-D1. (**b**–**e**) Quantification of the cell surface/total VSV-Plexin-D1 ratio in control growth cones and growth cones exposed to Sema3E (10 min of treatment) in the presence or absence of dynasore, Pitstop 2 or Pitstop 2-negative control. Sema3E induced clathrin- and dynamin-dependent internalization of Plexin-D1; *n*=number of growth cones analysed per condition in three independent experiments. Data are represented as mean±s.e.m., ***P<0.0001 by the Mann–Whitney test. (**f**) Examples of growth cones from E15.5 Pir neurons showing low (Control) and high (+Sema3E) endocytosis of FLAG-Plexin-D1. (**g**) Quantification of endocytosed FLAG-Plexin-D1 in growth cones illustrated in (**f**). Results indicate endocytosed of Plexin-D1 after Sema3E treatment; *n*=number of growth cones analysed per condition. Data are presented as mean±s.e.m. and values are indicated in arbitrary units (a.u.) of fluorescence. ***P<0.0001 by the Mann–Whitney test. Scale bars, 10 μm. See also Supplementary Fig. 2.

We next sought to determine whether the Plexin-D1 receptor was internalized in growth cones. Although detectable, the levels of endogenous Plexin-D1 expression were too low to allow the determination of its subcellular location. Therefore, a recombinant human Plexin-D1 receptor was expressed in Pir neurons. Despite an increase of ∼60% in binding sites for Sema3E, Pir neurons overexpressing Plexin-D1 showed a similar level of collapse response to Sema3E as compared with nontransfected neurons (Supplementary Fig. 2a–c). The surface localization of exogenously expressed Plexin-D1 receptors was monitored by immunolabelling with an antibody against the extracellular domain of the human Plexin-D1 receptor, followed by cell permeabilization and labelling of the total human Plexin-D1 content. After 10 min of treatment with Sema3E, the ratio of surface/total Plexin-D1 dropped from 70% in unstimulated condition to 28% (Fig. 2a,b). Pharmacological inhibitors of dynamin- and clathrin-dependent endocytosis suppressed Sema3E-induced removal of Plexin-D1 from the cell surface (Fig. 2c–e). We then confirmed endocytosis of Plexin-D1 using a live cell ‘antibody feeding' assay (Fig. 2f,g) and by showing an increased colocalization of Plexin-D1 with green fluorescent protein (GFP)-Rab5, a marker of early endosomes, in Sema3E-stimulated growth cones (Supplementary Fig. 2d,e). Finally, control experiments using Sema3B and Sema3C, which do not bind directly to Plexin-D1 but induce growth cone collapse of Pir neurons, did not show endocytosis of Plexin-D1 (Supplementary Fig. 2f–h). Together, these results reveal that Plexin-D1 undergoes endocytosis in growth cones before the peak in Sema3E-induced collapse.

### PDZ-dependent sorting of Plexin-D1 into recycling endosomes

**Figure 3:**
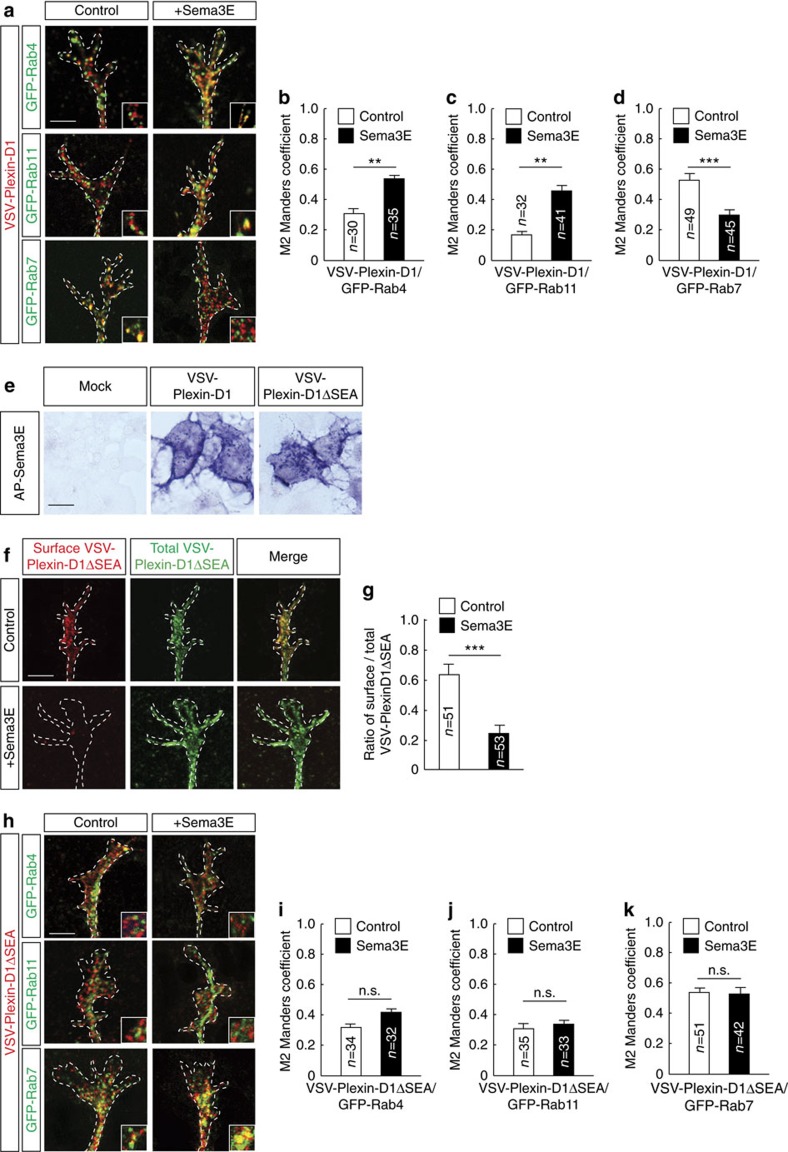
Sorting of Plexin-D1 into recycling pathways requires its SEA PDZ-domain-binding motif. (**a**) Colocalization of VSV-Plexin-D1 (red) and different GFP-tagged Rab proteins (green) in cultured E15.5 Pir neurons treated or not treated with Sema3E (10 min). (**b**–**d**) Graphs showing the Manders colocalization coefficients for the fraction of VSV-Plexin-D1 colocalized with GFP-Rab4, GFP-Rab11 or GFP-Rab7 in the presence or absence of Sema3E treatment (10 min). Ligand-activated Plexin-D1 receptors were directed to recycling endosomes; *n*=number of growth cones analysed per condition in three independent experiments. Data are represented as mean±s.e.m., **P<0.01, ***P<0.0001 by the Mann–Whitney test. (**e**) Alkaline phosphatase (AP)-tagged Sema3E binds equally well to COS7 cells expressing VSV-Plexin-D1 or VSV-Plexin-D1ΔSEA. No binding is observed on mock-transfected COS7 cells. (**f**) Examples of growth cones from E15.5 Pir neurons showing cell surface localization (Control) and internalization (+Sema3E) of VSV-Plexin-D1ΔSEA. (**g**) Quantification of the cell surface/total VSV-Plexin-D1ΔSEA ratio in control growth cones and growth cones exposed to Sema3E (10 min). Plexin-D1 lacking the SEA motif was internalized in growth cones in response to Sema3E ligand activation; *n*=number of growth cones analysed per condition in three independent experiments. Data are represented as mean±s.e.m., ***P<0.0001 by the Mann–Whitney test. (**h**) Colocalization of VSV-Plexin-D1ΔSEA (red) with different GFP-Rab proteins (green) in cultured E15.5 Pir neurons with or without Sema3E treatment (10 min). (**i**–**k**) Graphs showing the Manders colocalization coefficient for the fraction of VSV-Plexin-D1ΔSEA colocalized with GFP-Rab4, GFP-Rab11 or GFP-Rab7 with or without Sema3E treatment (10 min). Ligand-activated Plexin-D1ΔSEA was missorted to late endosomes; *n*=number of growth cones analysed per condition in three independent experiments. Data are represented as mean±s.e.m. No statistical difference was found between conditions using the Mann–Whitney test. Scale bars, 10 μm. See also Supplementary Fig. 2.

We next investigated the intracellular fate of internalized Plexin-D1. In the absence of ligand, a small amount of Plexin-D1 receptors colocalized intracellularly with GFP-Rab7, a marker of late endosomes (Fig. 3a,d). In contrast, 10 min of stimulation with Sema3E enhanced the sorting of the Plexin-D1 receptor to Rab4 and Rab11 endosomes that function in rapid and slow recycling, respectively (Fig. 3a–c). The efficient targeting of cargo proteins to recycling endosomes often requires the presence of specific sorting motifs, such as C-terminal PDZ domain-interacting sequences^19^. Because Plexin-D1 harbours a class I PDZ-domain-binding motif (serine–glutamate–alanine (SEA)), we investigated the role of this sequence in receptor endocytosis and post-endocytic sorting. Sema3E was able to bind to cells expressing a Plexin-D1 receptor lacking the SEA motif (Plexin-D1ΔSEA) (Fig. 3e and Supplementary Fig. 2a,b) and to trigger internalization of the mutant receptor in growth cones (Fig. 3f,g), but not a collapse response (Supplementary Fig. 2c). Like the wild-type receptor, under basal conditions, the Plexin-D1ΔSEA receptor residing intracellularly mainly distributed in late endosomes (Fig. 3h–k). However, after Sema3E stimulation, internalized wild-type and mutant receptors diverged in their post-endocytic sorting, as Plexin-D1ΔSEA remained in GFP-Rab7 late endosomes and did not accumulate in recycling endosomes (Fig. 3h–k). Thus, binding of Sema3E relocalized the Plexin-D1 receptor from the cell surface to intracellular recycling compartments via a sorting mechanism that requires its C-terminal PDZ-binding motif.

### GIPC1 controls Plexin-D1 receptor recycling

**Figure 4:**
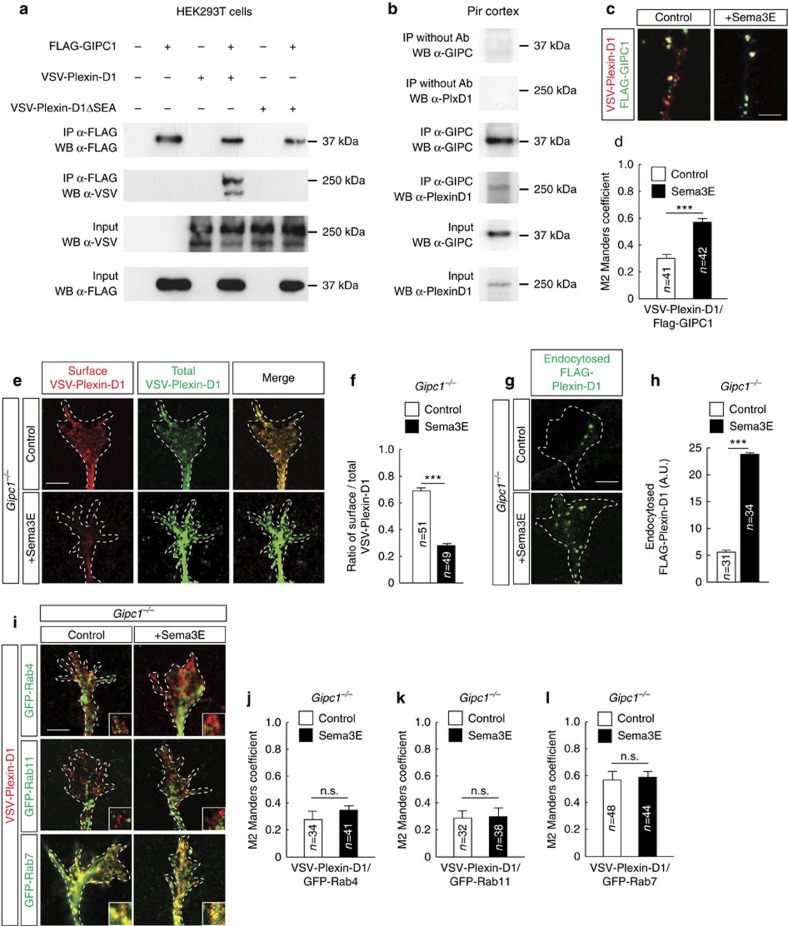
GIPC1 controls post-endocytic sorting of Plexin-D1. (**a**) HEK293T cells were transfected with FLAG-GIPC1, VSV-Plexin-D1 and VSV-Plexin-D1ΔSEA constructs. Proteins were immunoprecipitated (IP) from cell lysates and immunoblotted (WB) using the indicated antibodies. The C-terminal SEA motif of Plexin-D1 interacts with GIPC1. (**b**) Co-IP of endogenous GIPC1 and Plexin-D1 proteins from cell lysate of E15.5 Pir cortex. (**c**) Axons of E15.5 Pir neurons expressing FLAG-GIPC1 (green) and VSV-Plexin-D1 (red), with or without Sema3E treatment (10 min). (**d**) Graph showing the Manders colocalization coefficients for the fraction of VSV-Plexin-D1 colocalized with FLAG-GIPC1. Sema3E increased the colocalization of the two proteins; *n*=number of growth cones analysed per condition in three independent experiments. Data are represented as mean±s.e.m., ***P<0.001 by the Mann–Whitney test. (**e**) Growth cones of E15.5 *Gipc1*^*−/−*^ Pir neurons showing cell surface localization (Control) and internalization (+Sema3E) of VSV-Plexin-D1. (**f**) Quantification of the cell surface/total VSV-Plexin-D1 ratio in *Gipc1*^*−/−*^ growth cones. Sema3E induced internalization of Plexin-D1; *n*=number of growth cones analysed per condition in three independent experiments. Data are represented as mean±s.e.m., ***P<0.0001 by the Mann–Whitney test. (**g**) Examples of growth cones of E15.5 *Gipc1*^*−/−*^ Pir neurons showing low (Control) and high (+Sema3E) endocytosis of FLAG-Plexin-D1. (**h**) Quantification of endocytosed FLAG-Plexin-D1 in *Gipc1*^*−/−*^ growth cones. Sema3E induced internalization of Plexin-D1; *n*=number of growth cones analysed per condition. Data are presented as mean±s.e.m. and values are indicated in arbitrary units (A.U.) of fluorescence. ***P<0.0001 by the Mann–Whitney test. (**i**) Colocalization of VSV-Plexin-D1 (red) with different GFP-Rab proteins (green) in E15.5 *Gipc1*^*−/−*^ Pir neurons with or without Sema3E treatment (10 min). (**j**–**l**) Graphs show the Manders colocalization coefficients for the fraction of VSV-Plexin-D1 colocalized with GFP-Rab proteins in *Gipc1*^*−/−*^ growth cones. Ligand-activated Plexin-D1 was missorted to late endosomes; *n*=number of growth cones analysed per condition in three independent experiments. Data are represented as mean±s.e.m. No statistical difference was found between conditions using the Mann–Whitney test. Scale bars, 10 μm. See also Supplementary Figs 3 and 4.

One candidate molecule that may regulate sorting of Plexin-D1 is the PDZ domain-containing protein GIPC1 that can interact with receptors containing a C-terminal SEA motif^20^. The interaction between Plexin-D1 and GIPC1 was confirmed in lysates of HEK293T cells coexpressing the two proteins and occurred in a ligand-independent fashion (Fig. 4a and Supplementary Fig. 4a). The SEA residues were shown to mediate this interaction, as Plexin-D1ΔSEA did not co-precipitate with GIPC1 (Fig. 4a and Supplementary Fig. 4a). Moreover, the binding between GIPC1 and Plexin-D1 was specific among other plexins, as we found no interaction between GIPC1 and the other family members (Plexins B1, B2 and B3) harbouring a C-terminus PDZ-binding site that is structurally distinct from that of Plexin-D1 (refs 21, 22) (Supplementary Figs 3a and 4b).

**Figure 5:**
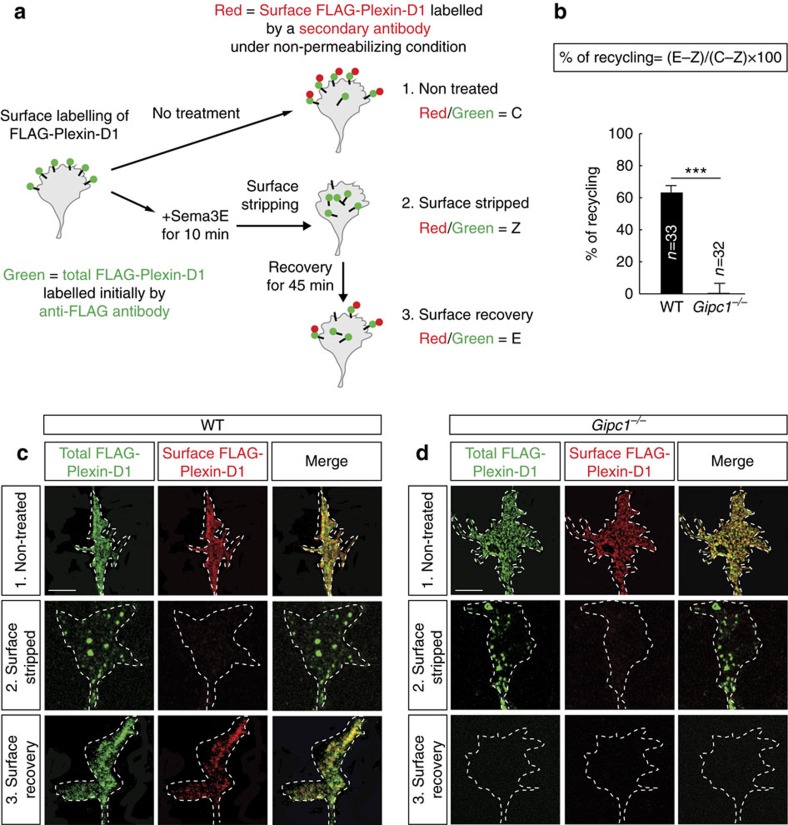
GIPC1 controls Plexin-D1 receptor recycling to the plasma membrane. (**a**) Schematic of the quantitative receptor recycling assay. (**b**) Quantification of the percentage of FLAG-Plexin-D1 recycling to the growth cone surface of wild-type (WT) or *Gipc1^−/−^* E15.5 Pir neurons. GIPC1 is required for recycling of internalized Plexin-D1 to the plasma membrane; *n*=number of growth cones analysed per condition. Data are represented as mean±s.e.m., ***P<0.0001 by the Mann–Whitney test. (**c**,**d**) Representative confocal fluorescence images of FLAG-Plexin-D1 recycling assay in growth cones of WT (**c**) and *Gipc*^*−/−*^ (**d**) E15.5 Pir neurons. Scale bars, 10 μm.

*Gipc1* mRNA was ubiquitously expressed in the developing mouse brain (Supplementary Fig. 3b) and interaction between endogenous Plexin-D1 and GIPC1 proteins was confirmed by co-immunoprecipitation of the complex from lysate of Pir cortex (Fig. 4b and Supplementary Fig. 4c). In cultured Pir neurons, GIPC1 protein was present along the length of the axons and in growth cones (Supplementary Fig. 3c,d) where it was enriched in Rab5, Rab4 and Rab11 endosomes and almost absent in Rab7 endosomes (Supplementary Fig. 3e–i). Some colocalization between Plexin-D1 and GIPC1 was observed in growing growth cones that was enhanced by stimulation with Sema3E (Fig. 4c,d), indicating that Sema3E is required to activate the plasma membrane-to-endosome traffic of Plexin-D1 and bring the two proteins in close proximity in early and/or recycling compartments of the endocytic pathway. We next determined the trafficking route of the wild-type Plexin-D1 receptor exogenously expressed in Pir neurons of *Gipc1*-deficient mouse embryos. GIPC1 depletion did not affect expression or surface localization of Plexin-D1 (Supplementary Fig. 3b,j–m) that was robustly internalized in growth cones within 10 min of application of Sema3E (Fig. 4e–h). However, the ligand-activated receptor was preferentially trafficked to the Rab7 endosomal compartment (Fig. 4i–l), similar to our observation for the mutant Plexin-D1ΔSEA receptor. We then examined the recycling of Plexin-D1 from endosomes to the growth cone surface using a previously described assay^23^ (Fig. 5a). In wild-type Pir neurons, 45 min after stimulation with Sema3E, 63% of internalized Plexin-D1 receptors have been recycled back to the surface of the growth cones (Fig. 5b,c). In contrast, in *Gipc1*-deficient neurons, the receptors were no longer recycled back to the growth cone surface (Fig. 5b,d). Furthermore, the fluorescent signal for internalized receptors had disappeared from the growth cones (Fig. 5d, no green signal in condition 3), indicating that the receptors have been degraded or transported to other location in the cell. Together, these results indicate a role for GIPC1 as an adaptor protein mediating PDZ-directed sorting of Plexin-D1 into the recycling pathway without affecting the initial step of receptor endocytosis.

### GIPC1 regulates growth cone response to Sema3E repulsion

**Figure 6:**
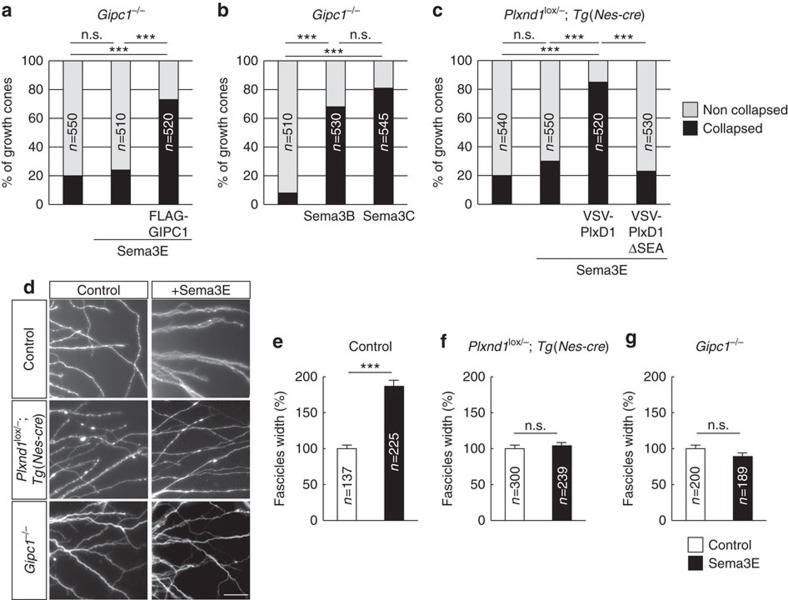
GIPC1 is required for axonal and growth cone response to Sema3E. (**a**–**c**) Quantification of the percentage of collapsed growth cones in response to 20 min of treatment with Sema3E, Sema3B or Sema3C in cultures of E15.5 Pir neurons of Gipc1^−/−^ or *Plxnd1*^*lox/−*^*;Tg(Nes-cre)* mutants. Sema3E-induced collapse required functional GIPC1 and the C-terminal SEA motif of Plexin-D1; *n*=number of growth cones analysed per condition in three independent experiments. The χ^2^ test, ***P<0.001. (**d**) Photomicrographs showing axons stained with calcein-AM growing out from E15.5 Pir explants of control, *Plxnd1*^*lox/−*^*;Tg(Nes-cre)* or *Gipc1*^*−/−*^ mutants, cultured for 2 days with or without Sema3E. (**e**–**g**) Quantification of the average fascicle width in response to Sema3E in cultures of control, *Plxnd1*^*lox/−*^*;Tg(Nes-cre)* or *Gipc1*^*−/−*^ mutant explants. Sema3E-induced fasciculation required expression of Plexin-D1 and GIPC1 in axons; *n*=number of fascicles measured per condition in three independent experiments. Data are shown as mean±s.e.m. and are normalized to the values obtained in unstimulated conditions. ***P<0.001, by the Mann–Whitney test. Scale bar, 50 μm.

Given that GIPC1 regulates the intracellular sorting, but not internalization, of Plexin-D1, we sought to address whether modulating GIPC1 function would affect growth cone responses to Sema3E. We found that Pir neurons from *Gipc1*^*−/−*^ mutant embryos failed to collapse upon Sema3E exposure (Fig. 6a). The collapse response was restored after the reintroduction of GIPC1 protein (Fig. 6a). Regulating growth cone repulsion was not a general function of GIPC1, however, as it was not required for the collapsing activity of Sema3B and Sema3C on Pir neurons (Fig. 6b). To test whether the interaction of GIPC1 to Plexin-D1 was directly required for the response to Sema3E, we expressed either the wild-type Plexin-D1 receptor or the mutant receptor missing the SEA motif in neurons from mouse embryos lacking endogenous Plexin-D1 (*Plxnd1*^*lox/−*^*;Tg(Nes-cre)* mice; Supplementary Fig. 3j). Unlike the wild-type receptor, Plexin-D1ΔSEA was unable to mediate Sema3E-induced collapse (Fig. 6c). Finally, we found that *Gipc1* was also required for Sema3E to cause Plexin-D1-dependent fasciculation of Pir axons (Fig. 6d–g). Together, these results indicate that downstream of endocytosis, the proper endosomal sorting of Plexin-D1 is required to trigger a repulsive cellular response to Sema3E.

### GIPC1 is required for Sema3E-induced inhibition of R-ras

**Figure 7:**
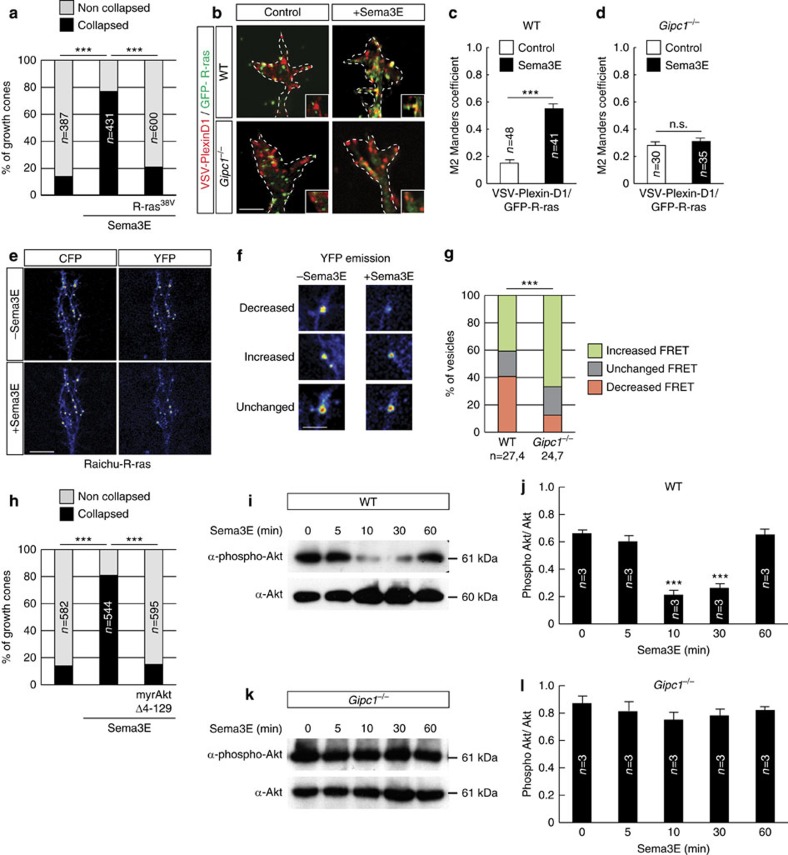
Impaired signal transduction in neurons lacking GIPC1. (**a**) Percentage of collapsed growth cones of E15.5 Pir neurons in response to Sema3E (20 min). The constitutively active form of R-ras (R-ras^38V^) abrogated the collapsing effect of Sema3E; *n*=number of growth cones per condition in three independent experiments. The χ^2^ test, ***P<0.0001. (**b**) Growth cones of E15.5 wild-type (WT) or *Gipc1*^*−/−*^ Pir neurons expressing GFP-R-ras and VSV-Plexin-D1, treated with or without Sema3E (10 min). (**c**,**d**) Graphs show Manders colocalization coefficients for the fraction of VSV-Plexin-D1 colocalized with GFP-R-ras. GIPC1 increased colocalization of Plexin-D1 and R-ras; *n*=number of growth cones per condition in three independent experiments. Data are represented as mean±s.e.m., ***P<0.001 by the Mann–Whitney test. (**e**) Expression of the Raichu-R-ras reporter in a E15.5 Pir neuron before and after the addition of Sema3E. CFP and YFP images are presented as pseudocolour images (red: high signal, blue: low signal). The CFP image (left) shows the distribution of R-ras on vesicles. The YFP signal (right) is proportional to the amount of GTP bound to R-ras. (**f**) Examples of changes in the YFP signal induced by exposure to Sema3E. (**g**) Percentage of vesicles displaying increased, decreased or unchanged FRET level. Sema3E-driven R-ras inhibition was reduced in *Gipc1*^*−/−*^ neurons; *n*=*x*,*y* where *x* indicates the number of vesicles and *y* the number of growth cones analysed. The χ^2^ test, ***P<0.0001. (**h**) Percentage of collapsed growth cones in response to Sema3E (20 min) in cultures of E15.5 Pir neurons. A constitutively active form of Akt (myrAkt Δ4–129) abrogated the collapsing effect of Sema3E; *n*=number of growth cones per condition in three independent experiments. The χ^2^ test, ***P<0.0001. (**i**,**k**) Phosphorylation of Akt in E15.5 WT or *Gipc1*^*−/−*^ Pir neurons stimulated with Sema3E (0 to 60 min). (**j**,**l**) Quantification of phospho-Akt levels. Sema3E-induced inhibition of Akt required GIPC1 function; *n*=number of experiments, data are mean±s.e.m., ***P<0.001 by the Mann–Whitney test. Scale bars, 10 μm (**b**,**e**), 2 μm (**f**). See also Supplementary Figs 5 and 6.

Based on the above results, we hypothesized that the localization of intracellular Plexin-D1 to endosomal recycling compartments may specifically regulate signal transduction events. Previous studies reported that the activation of Plexin-D1 results in the inhibition of R-ras, a member of the superfamily of small GTPases, via its GTPase-activating protein domain^24^,^25^. Consistent with this, introducing a constitutively active R-ras (R-ras^38V^) in Pir neurons prevented growth cone collapse induced by Sema3E (Fig. 7a). In heterologous cell lines, R-ras is enriched on vesicular structures positive for early endosomal/recycling markers^26^. In growth cones, R-ras similarly distributed to Rab4- and Rab11-positive endosomes and much less in Rab7 endosomes (Supplementary Fig. 5a,b). In the absence of Sema3E ligand, little colocalization was observed between R-ras and Plexin-D1 that then constitutively traffics through Rab7 compartments (Fig. 7b,c). However, colocalization between R-ras and Plexin-D1 increased significantly after the application of Sema3E (Fig. 7b,c). Finally, little colocalization between R-ras and Plexin-D1 was observed in growth cones lacking GIPC1, even after stimulation with Sema3E (Fig. 7b,d). Thus, GIPC1-dependent sorting of Plexin-D1 to specific endosomal compartments may promote a functional interaction with R-ras, a key component of the signal transduction machinery downstream of Sema3E.

To directly test whether Plexin-D1 inhibits R-ras at the level of endosomes, we used a Förster resonance energy transfer (FRET)-based biosensor for R-ras, called Raichu-R-ras, that allows a direct measurement of activity change of this protein in living cells^26^. In Pir neurons growing on a laminin/poly-lysine substrate, R-ras activity was high in vesicular structures within axonal growth cones (Fig. 7e). This is consistent with previous studies that have implicated R-ras in mediating integrin-dependent neurite outgrowth on laminin^27^,^28^. Currently, however, the upstream pathway that positively regulates R-ras activity is not known. Within 3–9 min after the addition of Sema3E, the FRET signal decreased on a portion of the vesicles (40.7%; Fig. 7e–g), indicating the inactivation of R-ras presumably in the recycling endosomes that traffic the activated Plexin-D1 receptor. In other vesicles, FRET signals increased (40.8%) or remained unchanged (18.5%; Fig. 7e–g). By contrast, in *Gipc1*^*−/−*^ growth cones, only 12.5% of the vesicles showed decreased R-ras activity after stimulation with Sema3E, and the large majority displayed increased or unchanged FRET signals (66.7% and 20.8%, respectively; Fig. 7g). These data indicate that GIPC1, by bringing into close proximity ligand-activated Plexin-D1 and active R-ras, controls Sema3E-dependent inhibition of R-ras on endosomes.

We further investigated whether the reduced inactivation of R-ras in the absence of GIPC1 affects downstream signalling. R-ras is a positive regulator of the PI3K/Akt pathway^29^. Expression of a constitutively active form of Akt (myrAkt Δ4–129) prevented the repulsive response to Sema3E (Fig. 7h), suggesting that Akt inhibition is required for Sema3E signalling. Indeed, we observed a marked decrease in the phosphorylation of Akt at S473 in lysates of Pir neurons stimulated for 10 min with Sema3E (Fig. 7i,j and Supplementary Fig. 6a). This process was inhibited in dynasore-treated neurons (Supplementary Fig. 5c,d) and in *Gipc1*^*−/−*^ neurons (Fig. 7k,l and Supplementary Fig. 6b). Together, these data are consistent with the idea that inhibition of the R-ras/PI3K/Akt signalling cascade through the Plexin-D1 receptor is dependent on GIPC1-mediated post-endocytic sorting of the receptor into recycling pathways.

### *Plxnd1* and *Gipc1* cooperate for axon tract formation *in vivo*

**Figure 8:**
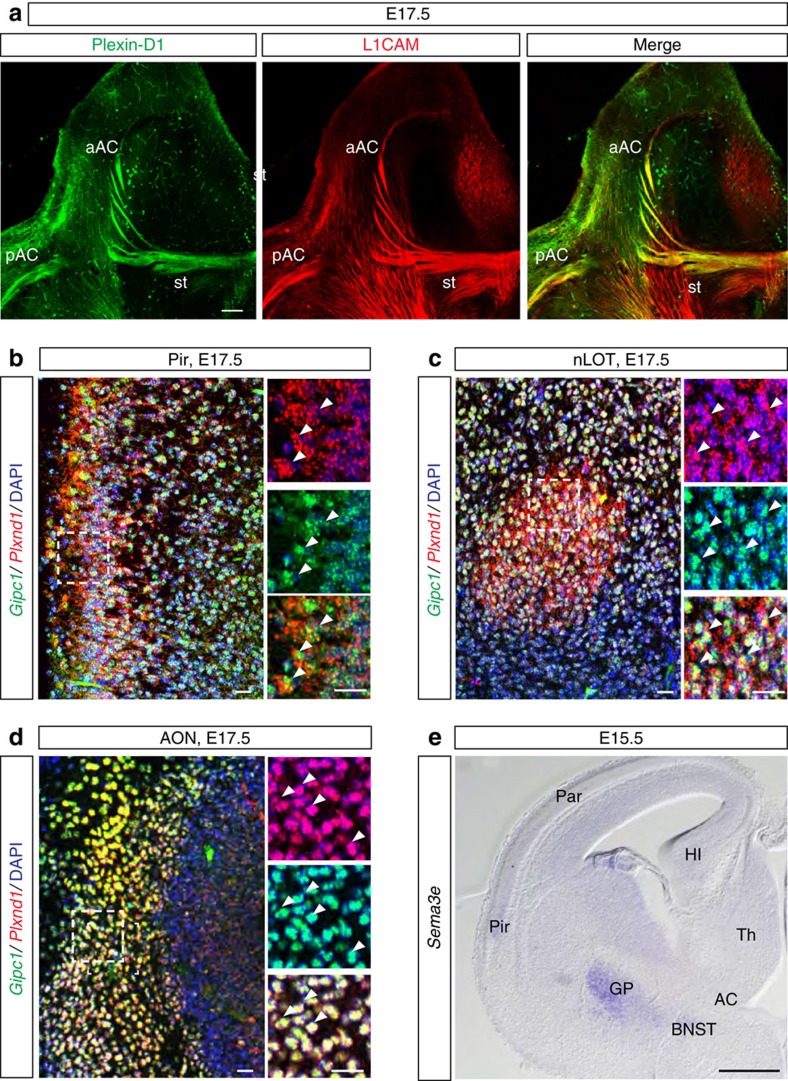
Plexin-D1 and GIPC1 are coexpressed in neurons of the AC. (**a**) Immunolabelling of Plexin-D1 (green) and L1CAM (red) in horizontal sections of E17.5 brain. Plexin-D1 is expressed on the three branches of the AC. (**b**–**d**) Fluorescent RNA *in situ* hybridization for *Gipc1* (green) and *Plxnd1* (red) on coronal sections of E17.5 wild-type mouse brain. *Gipc1* and *Plxnd1* mRNA are coexpressed in the Pir cortex (**b**), nLOT (**c**) and AON (**d**). (**e**) Coronal sections of E15.5 wild-type mouse brains were hybridized with an RNA probe for *Sema3e*. Strong signal was seen in the GP and BNST. aAC, anterior limb of the AC; AON, anterior olfactory nucleus; BNST, bed nucleus of the stria terminalis; GP, globus pallidus; HI, hippocampus; nLOT, nucleus of the lateral olfactory tract; pAC, posterior limb of the AC; Par, parietal cortex; Pir, piriform cortex; st, stria terminalis; Th, thalamus. Scale bars, 300 μm (**a**), 50 μm (**e**) and 20 μm (**b**–**d**).

**Figure 9:**
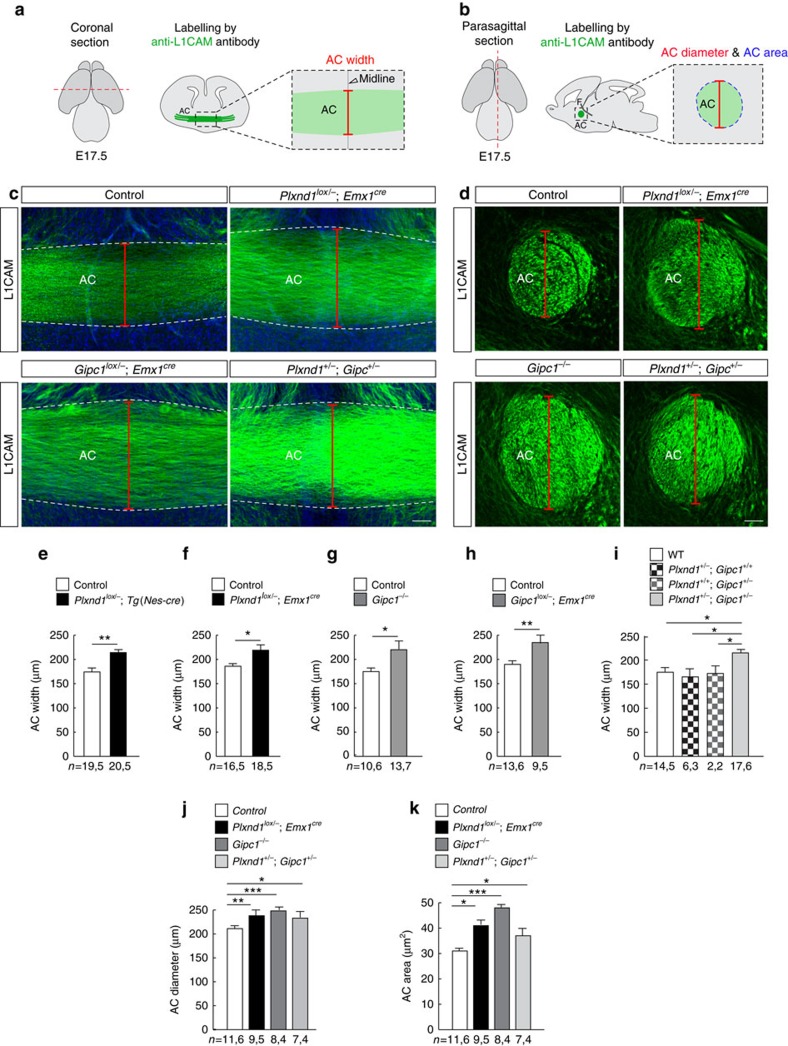
*Plxnd1* and *Gipc1* genetically interact to regulate the development of the AC. (**a**,**b**) Schematic illustrating the section planes and measurement methods for analysis of the AC. (**c**,**d**) Representative L1CAM-stained AC in coronal sections (**c**) and parasagittal sections at the level of the fornix (F) (**d**) of E17.5 brains from control, *Plxnd1*^*lox/−*^*;Emx1*^*cre*^ mutants, *Gipc1*^*lox/−*^*;Emx1*^*cre*^ mutants and *Plxnd1*^*+/*^*, Gipc1*^*+/−*^ double-heterozygous embryos. (**e**–**k**) Quantification of AC width (**e**–**i**), diameter (**j**) and cross-sectional area (**k**) in E17.5 brains from control mice, conditional *Plxnd1*^*lox/−*^*;Tg(Nes-cre)* and *Plxnd1*^*lox/−*^*;Emx1*^*cre*^ mutants, null *Gipc1*^*−/−*^ and conditional *Gipc1*^*lox/−*^*;Emx1*^*cre*^ mutants, *Plxnd1* and *Gipc1* single- (*Plxnd1*^+/−^, *Gipc1*^+/+^ and *Plxnd1*^+/+^, *Gipc1*^+/−^) and double-heterozygous (*Plxnd1*^*+/−*^*, Gipc1*^*+/−*^) mutants. Mice lacking Plexin-D1 and/or GIPC1 developed an enlarged AC. Data are shown as mean±s.e.m., *n*=*x*,*y* where *x* indicates the number of slices and *y* the number of mice analysed for each genotype. *P<0.05, **P<0.01, by the Mann–Whitney test (**e**–**h**), Kruskal–Wallis test (**i**–**k**). Scale bars, 50 μm (**c**), 40 μm (**d**). See also Supplementary Fig. 7. AC, anterior commissure. F, fornix.

Our observations indicate that GIPC1-regulated sorting of Plexin-D1 to recycling routes is required for receptor activity and signalling. How does this mechanism contribute to *in vivo* brain development? To address this question we first examined the requirement for *Plxnd1* in the establishment of the anterior commissure (AC), a tract containing the axons of the Pir neurons used in the *in vitro* analysis. In the developing mouse brain, Plexin-D1 protein was detected on the three branches of the AC (the anterior limb, the posterior limb and the commissural component of the *stria terminalis*; Fig. 8a), and *Plxnd1* and *Gipc1* mRNA were coexpressed by neurons located in the different fields of origin of the AC that include, in addition to the Pir cortex, the anterior olfactory nucleus and the nucleus of the lateral olfactory tract^30^,^31^ (Fig. 8b–d). *Sema3e* mRNA expression was detected in the globus pallidus, which is situated close to the AC, and in cells of the bed nucleus of the stria terminalis, which surround the AC at the midline (Fig. 8e). This expression profile suggests a role for Sema3E/Plexin-D1 signalling in channelling AC axons together. We tested this hypothesis by analysing the development of the AC in mice with conditional inactivation of *Plxnd1* in the nervous system (*Plxnd1*^*lox/−*^*;Tg(Nes-cre)* mice) or in forebrain glutamatergic neurons with a pallial origin (*Plxnd1*^*lox/−*^*;Emx1*^*cre*^ mice) that include the AC neurons but not the subpallium territory through which AC axons project. The AC was labelled with an anti-L1CAM antibody on coronal and sagittal sections of E17.5 brains (Fig. 9a–d). In both genotypes, the AC appeared enlarged in regions close to the brain midline (Fig. 9c–f,j,k), despite normal brain size (Supplementary Fig. 7a). This enlargement was observed from E14.5, when the first commissural axons crossed the brain midline, and persisted at least until postnatal day (P) 30, after the development of the AC has finished (Supplementary Fig. 7b,c). We verified that the number of projection neurons in the Pir, anterior olfactory nucleus and nucleus of the lateral olfactory tract did not vary in *Plxnd1*^*lox/−*^*;Tg(Nes-cre)* embryos compared with controls (Supplementary Fig. 7d–g), indicating that *Plxnd1* deletion did not affect the generation and specification of neurons. In some contexts, AC hyperplasia might serve as a compensatory mechanism for the congenital absence of another cortical commissure, the corpus callosum^31^,^32^,^33^. However, no sign of corpus callosum dysgenesis or misrouting of neocortical axons towards the AC was found in *Plxnd1*^*lox/−*^*;Tg(Nes-cre)* embryos (Supplementary Fig. 7h,i). Together, these data indicate that Plexin-D1 acts cell autonomously to regulate the development of the AC.

We next asked whether GIPC1 might contribute to Plexin-D1 function in this system. In E17.5 embryos with constitutive (*Gipc1*^*−/−*^) or conditional deletion of *Gipc1* in neurons of the AC (*Gipc1*^*lox/−*^*;Emx1*^*cre*^), the AC was larger than in control embryos (Fig. 9c,d,g–k; Supplementary Fig. 7a). Last, animals harbouring double heterozygous mutations for *Plxnd1* and *Gipc1* also displayed a significant increase in AC size that was not observed in either single *Plxnd1*^*−/+*^ or *Gipc1*^*−/+*^ heterozygous mutants (Fig. 9c,d,i–k; Supplementary Fig. 7a). Together, these data demonstrate that GIPC1 together with Plexin-D1 play a critical role in the formation of a major axon tract from the cerebral cortex.

**Figure 10:**
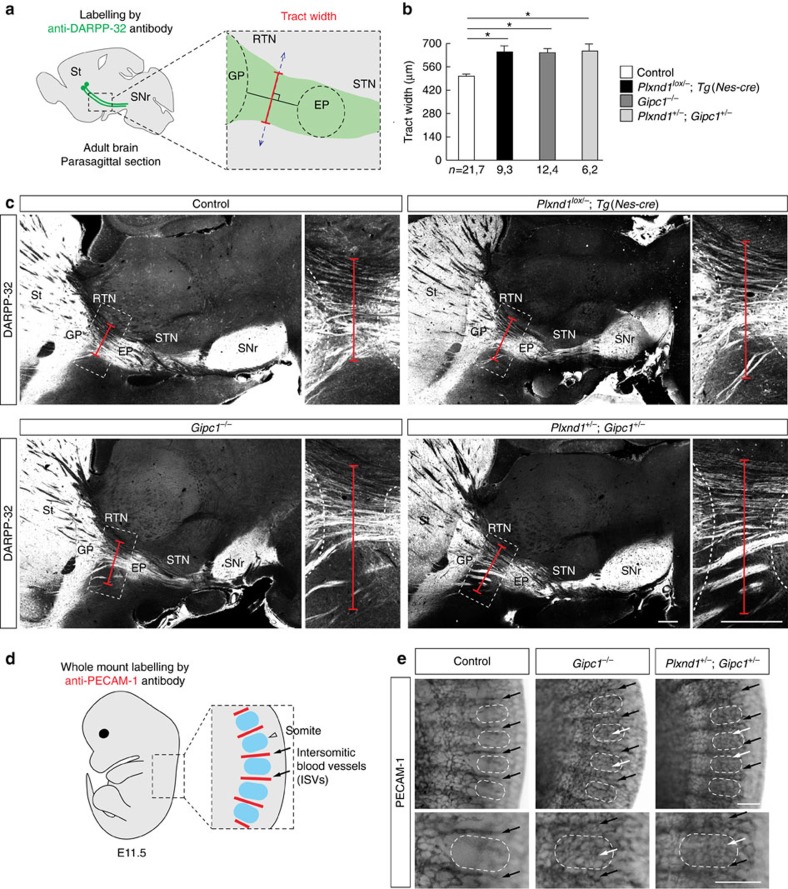
*Plxnd1* and *Gipc1* genetically interact to regulate the development of the striatonigral pathway and intersomitic blood vessels. (**a**) Schematic representing the region of interest in parasagittal sections of adult mouse brains and the quantification method for analysis of the striatonigral tract. The width (red segment) of the striatonigral tract (green) was measured at equal distance between the border of the globus pallidus (GP) and the entopeduncular nucleus (EP) on parasagittal sections, along a line perpendicular (dashed blue line) to the main orientation of the tract. (**b**) Quantification of the width of the striatonigral tract in control, *Plxnd1*^*lox/−*^*;Tg(Nes-cre)*, *Gipc1*^*−/−*^ or double-heterozygous *Plxnd1*^*+/−*^*, Gipc1*^*+/−*^ mutant brains. Mice lacking Plexin-D1 or GIPC1 and double heterozygous mice displayed an enlargement of the striatonigral axon tract. Data are shown as mean±s.e.m., *n*=*x*,*y* where *x* indicates the number of slices and *y* the number of mice analysed for each genotype. *P<0.05, by the Kruskal–Wallis test. (**c**) Representative DARPP-32-stained striatonigral projections in parasagittal sections of adult brains from control, *Plxnd1*^*lox/−;*^*Tg(Nes-cre)*, *Gipc1*^*−/−*^ or double-heterozygous *Plxnd1*^*+/−*^*, Gipc1*^*+/−*^ mutant mice. Red segments delineate the tract width. Onsets show high magnifications views of the tract (dashed boxes in main pictures). (**d**) Schematic drawing showing the region of interest for analysis of intersomitic blood vessels (ISVs). (**e**) Whole-mount PECAM-1 staining of E11.5 embryos from control (*n*=15 mice), *Gipc1*^*−/−*^ (*n*=6 mice) and double-heterozygous *Plxnd1*^*+/−*^*, Gipc1*^*+/−*^ mutants (*n*=5 mice). Dashed oval, somite; black arrow, ISV; white arrows, misguided ISV. Mice lacking GIPC1 and double heterozygous mice show disruption of the ISV vascular pattern. EP, entopeduncular nucleus; GP, globus pallidus; ISVs, intersomitic blood vessels; RTN, reticular thalamic nucleus; SNr, substantia nigra; St: striatum; STN, subthalamic nucleus. Scale bars, 500 μm. See also Supplementary Fig. 8.

To further explore how general is the requirement for GIPC1 in the development of Plexin-D1-expressing axonal projections, we performed additional characterization of the *Plxnd1* and *Gipc1* mutants and compared the results against known phenotypes of *Sema3e* gene alterations. Previous studies identified a role for Sema3E expression in the globus pallidus and reticular thalamic nucleus in the development of the striatonigral pathway^16^,^34^. Labelling of striatal projections with an anti-DARPP-32 (Dopamine- and cAMP-regulated neuronal phosphoprotein 32) antibody in brains of adult *Plxnd1*^*lox/−*^*;Tg(Nes-cre)*, *Gipc1*^*−/−*^ and double *Plxnd1*^*−/+*^;*Gipc1*^*−/+*^ heterozygous mutant mice revealed in each mutant genotype an enlargement of the striatonigral tract (Fig. 10a–c). Altogether, these data demonstrate in two distinct populations of neurons that Plexin-D1 and GIPC1 interact in the same molecular pathway to properly control axon projection patterns.

### Sema3E promotes axon growth independently of GIPC1

In addition to its repulsive activity, Sema3E can also attract and promote the growth of efferent axons of the subiculum^16^. In E17.5 mouse embryos lacking either *Sema3e* or *Plxnd1*, very few axons reached the postcommissural part of the fornix tract^16^. In this particular context, Plexin-D1 is required on axons for Sema3E ligand binding but not for signal transduction that is initiated by the co-receptor vascular endothelial growth factor receptor-2 (VEGFR-2)^35^. If Plexin-D1 does not directly convey signal, then *Gipc1* loss of function would not be expected to affect fornix development. Indeed, we found that the postcommissural fornix was formed normally in *Gipc1* null mutants and in double heterozygous mutants for *Plxnd1* and *Gipc1* (Supplementary Fig. 8a–d). This independence for GIPC1 function was confirmed *in vitro*, as shown by the ability of Sema3E to stimulate elongation of subicular axons lacking *Gipc1* or expressing the Plexin-D1ΔSEA mutant receptor (Supplementary Fig. 8e–g). Thus, GIPC1 is specifically required during brain development for controlling Sema3E-dependent axonal repulsion, but not elongation.

### *Plxnd1* and *Gipc1* cooperate during vascular patterning

Finally, we speculated that the GIPC1-dependent regulation of the post-endocytic sorting and signalling of the Plexin-D1 receptor might be a general mechanism that operates in other cell types outside the nervous system. In the trunk region of mouse embryos, expression of *Sema3e* in somites repels the growth of adjacent intersomitic blood vessels (ISVs) that express Plexin-D1 (ref. 14). Here we found that in E11.5 *Gipc1*^−/−^ embryos, ISVs labelled with anti-PECAM-1 (platelet-endothelial cell adhesion molecule-1) antibody ectopically extended throughout the somites, resulting in a loss of their normal segmental organization (Fig. 10d,e). This phenotype was similar to that reported in mice lacking *Plxnd1* (ref. 14). Moreover, double *Plxnd1*^+/−^, *Gipc1*^+/−^ heterozygous mutants showed a similar disturbed pattern of ISV organization (Fig. 10e). These data support an extended role for the adaptor protein GIPC1 in controlling repulsive Sema3E/Plexin-D1 signalling during patterning of the developing mouse vasculature.

## Discussion

This study uncovers a specific role for the adaptor protein GIPC1 in coupling endosomal sorting of the Plexin-D1 receptor to the initiation of repulsive guidance signalling. Because ligand-induced internalization has been reported for several members of the plexin family^11^,^12^,^36^, our results raise the question of how general the regulation of plexin signalling by active intracellular trafficking may be. The cytoplasmic Ras GAP domains are highly conserved among all plexin subfamilies and, so far, three small GTPases of the Ras family, R-ras, M-ras and Rap1, have been identified as targets of plexin activity^37^,^38^,^39^. Although it remains controversial which of these Ras family proteins is the most relevant for semaphorin-mediated repulsion, they have all been shown to reside primarily on endosomes. However, they exhibit different subcellular localizations: whereas activated R-ras signals at the membrane of recycling endosomes, Rap1 activity is mainly associated with late endosomes^40^,^41^. Thus, an attractive hypothesis is that receptor endocytosis is a general mechanism by which plexins inhibit the activation of Ras proteins on endosomes and that some specificity towards the different Ras protein isoforms may be achieved through spatial restriction by post-endocytic sorting into distinct intracellular trafficking routes.

The question of how the Plexin-D1 receptor uses GIPC1 to enter the recycling pathways remains unanswered. One current model proposes that recycling depends on the capacity of internalized receptors to enter into specialized tubular microdomains of the early endosomes that undergo scission and transport to the cell surface via direct or slow indirect routes. A crucial element of the machinery that recruits cargo receptors into these endosomal tubules is a pentameric protein complex termed ‘retromer'^42^. Interestingly, a recent proteomic study of non-neuronal cells identified several plexins, including Plexin-D1, as cargo proteins of the retromer^43^. The retromer constitutes a central platform for the recruitment of a number of accessory proteins that aid in cargo sorting. Among these, the WASH (Wiskott-Aldrich syndrome protein and SCAR homologue) complex, an Arp2/3 activator, promotes the formation of branched actin filaments on endosomal tubules^44^. The molecular motors Myosin VI and Myosin Ic might employ these WASH complex-generated actin filaments to direct cargo proteins to endosomal tubules^44^. Interestingly, GIPC1 possesses a Myosin VI-binding site, and the two proteins have been shown to bind and regulate protein trafficking in a number of cell types^45^,^46^. However, expressing a dominant negative Myosin VI construct in Pir neurons did not impair axonal response to Sema3E (Supplementary Fig. 9), indicating that GIPC1 functions independently of Myosin VI. Further studies are needed to determine whether GIPC1 contributes to retromer-dependent trafficking events by serving as an adaptor protein linking receptors to components of the retromer, WASH complex and/or to actin motors.

So far, there have been few tests of the significance of endocytic trafficking of guidance receptors in the establishment of neural circuits *in vivo*. Blocking endocytic removal of guidance receptors from the cell surface (including Eph, TrkA and Robo receptors) has been shown to cause defects in axon pathfinding consistent with defective receptor signalling^9^,^47^,^48^. The importance of the intracellular machinery by which neurons degrade or recycle proteins after endocytosis has also been reported^49^,^50^,^51^. However, how inappropriate sorting of internalized guidance receptors to either fate leads to defects in axonal tract formation had not been tested. The present study provides *in vivo* evidence that the sorting adaptor GIPC1 is required for the development of two major projections from the cortex and striatum in the mouse brain, and plays additional function in guiding blood vessel patterning in the trunk.

In endothelial cells, GIPC1 has been previously reported to interact with the C-terminus SEA motif of the guidance receptor Neuropilin-1 (Nrp1) to modulate responses to semaphorin ligands and VEGF^52^,^53^. However, this mechanism is unlikely to be involved in the developing ISVs, since mice lacking the intracellular domain of Nrp1, and therefore the GIPC1-binding motif, do not have obvious defects in angiogenesis of ISVs^54^. It is also unlikely that GIPC1 mediates Nrp1-Sema or Nrp1-VEGF signalling in the nervous system because *Gipc1*-deficient neurons responded normally to the *in vitro* collapsing activity of several neuropilin-binding semaphorins, including Sema3B and Sema3C. Rather, the neuronal and vascular defects that we observed in *Gipc1* mutants likely results from Plexin-D1 receptor misrouting and the consequent loss of repulsive signalling, as further supported by the genetic interaction between *Gipc1* and *Plxnd1*.

Recently, the Drosophila GIPC homologue (dGIPC) has been linked to repulsive semaphorin/plexin signalling during motor axon guidance^55^. In contrast to the present model, dGIPC did not directly interact with the plexin receptor but served to target or stabilize the plexin-associated receptor guanylyl cyclase Gyc76c at the cell surface to promote the semaphorin-induced production of cGMP^55^,^56^. The functional consequences of regulated receptor trafficking for the physiological and pathological development of brain connectivity will continue to emerge as the intracellular trafficking routes of more guidance receptors and the mechanisms regulating their post-endocytic sorting are characterized.

## Methods

### Outcome assessment

All analyses were performed with the experimenter blind to genotypes and treatment conditions.

### Reagents and antibodies

The following reagents were used: mouse Sema3B-Fc (10 nM, R&D Systems), mouse Sema3C-Fc (10 nM, R&D Systems), mouse Sema3E-Fc (10 nM, R&D Systems), Texas Red-X phalloidin (1:50, Life Technologies), calcein-AM (1 μM, Sigma-Aldrich), Pitstop 2 and Pitstop 2-negative control (10 μM, Abcam) and dynasore (80 μM, Sigma-Aldrich). Antibodies include goat anti-PlexinD1 (1:100, R&D Systems, Cat. No. AF4160), anti-PlexinD1 (1:200, Abcam, Cat. No. 93234), rat anti-L1CAM (1:500, Millipore, Cat. No. MAB5272), rabbit anti-GIPC1 (1:150, GeneTex, Cat. No. GTX78211), mouse anti-α-tubulin (1:2,000, Sigma-Aldrich, Cat. No. T9026), rabbit anti-GFP (1:500, Torey Pines Biolabs, Cat. No. TP401), chicken anti-GFP (1:500, Aves Labs, Cat. No. 1020), rabbit anti-TBR1 (1:500, Abcam, Cat. No. 31940), rat anti-CTIP2 (1:250, Abcam, Cat. No. 18465), mouse anti-Myc (1:300, Sigma-Aldrich, Cat. No. M4439), horseradish peroxidase (HRP)-conjugated anti-VSV-G tag antibody (1:3,000, Abcam, Cat. No. 3556), mouse anti-FLAG (1:800, Clone M1, Sigma-Aldrich, Cat. No. F3040), rabbit anti-FLAG (1:4,000, Sigma-Aldrich, Cat. No. F7425), rabbit anti-phospho-S473-Akt (1:1,000, Promega, Cat. No. G744A), rabbit anti-Akt (1:1,000, Cell Signaling, Cat. No. 9272), rat anti-PECAM-1 (1:150, BD Pharmingen, Cat. No. 553370) and goat anti-DARPP32 (1:200, Santa Cruz, Cat. No. 8483). The Alexa Fluor 488 Protein Labelling Kit (Invitrogen) was used to label the mouse anti-FLAG antibody. We used appropriate secondary antibodies that were either conjugated to HRP (Vector Laboratories) or fluorescently labelled (Life Technologies).

### Plasmids

Expression constructs encoding VSV-tagged human Plexin-D1 and mouse AP-Sema3E-6xHis (AP-Sema3E) were reported previously^16^,^35^. The Plexin-D1 mutant lacking the SEA motif (VSV-Plexin-D1ΔSEA) was generated by PCR-mediated mutagenesis from the VSV-Plexin-D1 expression vector using the following primers: 5′-GTGCTACTAGTAGGCCTGAGACACATGGAGAGTTGGTCAGGC-3′ and 5′-CAGGCCTACTAGTAGCACTCGTAGATGTTGTCCTCCATCAAAGCC-3′. The VSV-PlexinB1, VSV-PlexinB2 and VSV-PlexinB3 expression vectors were gifts from A. Püschel^57^. The GFP-Sema3E and FLAG-Plexin-D1 plasmid were generated commercially by GeneCust. The FLAG-GIPC1 construct was a gift from M.G. Farquhar's Lab^58^. The CLC-CFP construct was a gift of R. Jacob^59^. R-Ras^38V^ was a gift from A. Hall's lab^60^, R-ras Raichu, pCXN2-5MycRab4, pCXN2-5MycRab5a and pCXN2-5MycRab11 were gifts of M. Matsuda^26^ and myrAkt Δ4–129 was a gift from D. Kaplan^61^.

### Animals

All animal procedures were conducted in accordance with the guidelines from the French Ministry of Agriculture (agreement number F1305521) and approved by the local ethics committee (C2EA-14 agreement 2015060510102024-V7 #1186). *Plxnd1* null and conditional *Plxnd1;Emx1*^*cre*^ mice have been reported previously^16^,^62^,^63^. *Plxnd1*^−/+^ mice were crossed with *Tg(Nes-Cre)* mice^64^ to generate *Plxnd1*^−/+^;*Tg(Nes-cre)* males. *Plxnd1*^−/+^;*Tg(Nes-cre)* (or *Plxnd1*^*−/+*^*;Emx1*^*cre*^) males were crossed to *Plxnd1*^*lox/lox*^ (ref. 65) females to generate *Plxnd1*^*lox/−*^*;Tg(Nes-cre) (*or *Plxnd1*^*lox/−*^*;Emx1*^*cre*^) mutants and littermate controls, including *Plxnd1*^*lox/+*^*;Tg(Nes-cre) (*or *Plxnd1*^*lox/+*^*;Emx1*^*cre*^), *Plxnd1*^*+/lox*^ and *Plxnd1*^*−/lox*^ mice. The control genotypes did not show significant differences and were pooled into a single group for this study. The genotype of the offspring was determined by PCR^16^,^62^. *Gipc1*^*−/−*^ mice were obtained by crossing the *Gipc1*^*lox/lox*^ (ref. 66) with a ubiquitous deleter cre [B6.C-Tg(CMV-cre)1Cgn/J]^67^ and crossed with *Emx1*^*cre*^ to get *Gipc1*^*−/+*^;*Emx1*^*cre*^ males. *Gipc1*^*−/+*^;*Emx1*^*cre*^ males were then crossed to *Gipc1*^*lox/lox*^ females to generate *Gipc1*^*lox/−*^;*Emx1*^*cre*^ mutants and the negative controls *Gipc1*^*lox/+*^;*Emx1*^*cre*^*, Gipc1*^*+/lox*^ and *Gipc1*^*−/lox*^ that were used in this analysis. The three control genotypes did not show significant differences and were pooled into a single group. The *Gipc1* floxed allele was genotyped with the following primer pair: lox*-*Fwd, 5′-AAGCAAAGGACAGTGCCAGT-3′ and lox-Rev, 5′-GGACCCACATACCTAGACTGC-3′; the *Gipc1* null allele was genotyped with the lox-Fwd primer and null-Rev, 5′-ACAACCTCCGAGCCTCATAA-3′. Transgenic mice expressing *GFP* under the control of the *Plxnd1* promoter [Tg(Plxnd1-EGFP)HF78Gsat/Mmucd] were purchased from the Mutant Mouse Resource Research Centers (MMRRC).

### Explant and dissociated neuronal cultures

Embryonic brains from wild-type CD1 mice, *Plxnd1* mutants, *Gipc1* mutants or control littermates were dissected to extract the Pir cortex at E15.5, the neocortex at E15.5 or the subiculum at E17.5. A detailed protocol for dissociated cultures and electroporation is available in Chauvet *et al*.^68^.

### Growth cone collapse assays

After 48 h in culture, dissociated E15.5 Pir neurons were incubated with recombinant Sema3B, Sema3C or Sema3E for 20 min at 37 °C, fixed in 4% paraformaldehyde (PFA), immunostained with mouse anti-tubulin antibody and labelled with Texas Red-X Phalloidin. Fluorescent-stained growth cones were imaged with a confocal microscope (Zeiss LSM 510 Meta) equipped with a 63 × -oil Plan-NEOFLUAR objective. Growth cones were scored as collapsed if their peripheral lamellipodia were absent, and if they had fewer than three filopodia or as non-collapsed. Data were pooled from three independent experiments and the percentages of collapsed and non-collapsed growth cones were calculated for each condition. The statistical significance of differences between conditions was evaluated using the χ^2^ test.

### Axonal growth assays

Dissociated neurons from E17.5 subiculum were cultured for 2–3 days *in vitro* in the presence or absence of Sema3E, fixed in 4% PFA and immunostained with mouse anti-tubulin antibody or rabbit anti-GFP antibody. Axonal length was quantified using the ImageJ plugin NeuronJ^16^. Data were pooled from three independent experiments and mean was calculated. Statistical significance of differences between means was evaluated using the Mann–Whitney test.

### Fasciculation assays

Explants of E15.5 Pir cortex grown for 48 h in the presence or absence of Sema3E were incubated with calcein-AM for 30 min at 37 °C. Fluorescent-stained explants were imaged with an inversed microscope (Zeiss AxioObserver D1) equipped with a 20 × Plan-NEOFLUAR objective. Quantification of axon fasciculation was performed by measuring fibre bundle thickness at 100 μm from the explant's edge. Data were pooled from three independent experiments and mean was calculated. Statistical significance of differences between means was evaluated using the Mann–Whitney test.

### Receptor internalization assays

After 48 h in culture, dissociated E15.5 Pir neurons expressing VSV-PlexinD1 or VSV-PlexinD1ΔSEA were incubated with Sema3B, Sema3C or Sema3E for 10 min at 37 °C and fixed in 4% PFA. Immunostaining with goat anti-Plexin-D1 antibody under non-permeabilizing conditions was performed to detect surface-expressed receptors, and total expression was detected after permeabilization with 0.1% Triton X-100. Fluorescent-stained growth cones were imaged with a confocal microscope (Zeiss LSM 510 Meta) equipped with a 63 × -oil Plan-NEOFLUAR objective. Parameters of time interval and gain setting on the camera were adjusted so that the brightest areas did not reach saturation. The immunofluorescence within the area of the growth cone was measured using ImageJ and the ratio of surface fluorescence to total fluorescence was calculated for each growth cone analysed. Data were pooled from three independent experiments and mean was calculated. Statistical significance of differences between means was evaluated using the Mann–Whitney test.

For antibody-feeding assay, neurons expressing FLAG-Plexin-D1 were surface labelled with Alexa Fluor 488-conjugated anti-FLAG antibody for 15 min at 37 °C and were incubated or not with Sema3E for 10 min at 37 °C. Anti-FLAG antibodies bound to noninternalized receptors were stripped from the cell surface by washing the cells quickly in phosphate-buffered saline lacking Ca2+ and Mg2+ supplemented with 0.04% EDTA, leaving behind only antibody bound to the internalized pool of receptors. Cells were fixed with 4% formaldehyde and 15% sucrose for 10 min at room temperature. Growth cones were imaged with a confocal microscope (Zeiss LSM 510 Meta) equipped with a 63 × -oil Plan-NEOFLUAR. Receptor endocytosis was quantified for each growth cone by measuring green fluorescence intensity using ImageJ. The mean fluorescence intensity was calculated and was expressed in arbitrary unit. The statistical significance of differences between means was evaluated using the Mann–Whitney test.

### Receptor intracellular trafficking and colocalization assays

After 48 h in culture, dissociated Pir neurons transfected with two different expression vectors were incubated with Sema3E for 10 min at 37 °C. Dual-colour immunostaining with the required primary antibodies were performed under permeabilized condition to detect coexpressed proteins. Growth cones were imaged with a confocal microscope (Zeiss LSM 510 Meta) equipped with a 63 × -oil Plan-NEOFLUAR objective. Parameters of time interval and gain setting on the camera were adjusted so that the brightest areas did not reach saturation. For each growth cone, the level of colocalization was measured by calculating the Manders coefficients in ImageJ software using the JACoP plug-in ref. 69. Data were pooled from three independent experiments and mean was calculated. The statistical significance of differences between means was evaluated using the Mann–Whitney test.

### Receptor recycling assay

This assay has been described in Choy *et al*.^23^. Briefly, neurons expressing FLAG-Plexin-D1 were surface labelled with Alexa Fluor 488-conjugated anti-FLAG antibody for 15 min at 37 °C, and were subjected to 3 sets of conditions in parallel as indicated: *Condition 1*, nontreated (C)—cells were fixed after 30 min of incubation in the absence of Sema3E and without a surface stripping step; *Condition 2*, surface stripped (Z)—cells were incubated with Sema3E for 10 min at 37 °C, followed by a EDTA stripping step to remove residual Alexa Fluor 488-conjugated anti-FLAG antibody from the cell surface by washing the cells quickly in phosphate-buffered saline lacking Ca2+ and Mg2+ supplemented with 0.04% EDTA, leaving behind only antibody bound to the internalized pool of receptors; and *Condition 3*, surface recovery (E)—EDTA-stripped cells as mentioned above were incubated for 45 min at 37 °C in fresh Neurobasal medium to let recycling occurred. For all 3 sets of conditions, cells were fixed with 4% formaldehyde and 15% sucrose for 10 min at room temperature under nonpermeabilizing condition, and stained with Alexa Fluor 568-conjugated anti-mouse secondary antibodies to label cell surface FLAG-Plexin-D1 receptors. Growth cones were imaged with a confocal microscope (Zeiss LSM 510 Meta) equipped with a 63 × -oil Plan-NEOFLUAR. Ratiometric image analysis was done using ImageJ by calculating the ratio of fluorescence intensity of nonpermeabilizing staining of cell surface FLAG-Plexin-D1 by a secondary antibody (red channel) to the overall intensity of FLAG-Plexin-D1 initially labelled with anti-FLAG antibody on the plasma membrane (green channel). The percentage of receptors recycled in individual cells was calculated from the red/green ratios from the 3 sets of conditions using the following formula: % Recycling=(E−Z)/(C−Z) × 100%. The average of percentage of recycling was calculated and statistical significance of differences between groups was evaluated using the Mann–Whitney test.

### Immunoprecipitation and western blotting

HEK293T cells (ATCC, mycoplasma free tested) were transfected with Flag-GIPC1 and/or equal amounts of VSV-PlexinD1, VSV-PlexinD1DSEA, VSV-PlexinB1, VSV-PlexinB2 or VSV-PlexinB3 expression vectors using Lipofectamine Plus (Invitrogen). At 2 days after transfection, the cell lysates were immunoprecipitated using ANTI-FLAG M2 Affinity Gel (Sigma-Aldrich) or VSV-G tag antibody Agarose. E15.5 Pir cortex cell lysate was immunoprecipitated with anti-GIPC1 antibody immobilized on protein A-Sepharose beads. Akt phosphorylation was evaluated on lysates of E15.5 Pir neurons cultured for 2 days and serum-starved for 2 h before Sema3E stimulation (from 0 to 30 min). Complexes and/or cell lysates were separated by electrophoresis and electrotransferred onto membranes (Immobilon-P, Millipore). Membranes were incubated with HRP-conjugated VSV-G tag, rabbit anti-FLAG, rabbit anti-GIPC1, rabbit anti-PlexinD1, anti-pS473 Akt or anti-Akt antibodies. After incubation with HRP-conjugated secondary antibodies, signals were detected with an enhanced chemiluminescence system (GE Healthcare). Akt activation was quantified by measuring the intensity of hybridized bands using ImageJ software and by calculating the phospho-Akt/Akt intensity ratio for each individual experiment. Ratios obtained from three independent experiments were averaged and statistical difference between means was evaluated by Mann–Whitney test. Images have been cropped for presentation. Full size images are presented in Supplementary Figs 4 and 6.

### Production of fusion proteins and binding assay

Conditioning media containing recombinant mouse AP-Sema3E and GFP-Sema3E proteins were obtained from transiently transfected HEK293T cells^68^. Quantification of GFP-Sema3E was performed using the GFP quantification kit (Biovision). AP-Sema3E binding experiments on COS7 cells (ATCC) were performed by conventional methods^16^. For binding experiments on neurons, after 10 min of treatment with 10 nM GFP-Sema3E, dissociated neurons were fixed in 4% PFA and immunostained with chicken anti-GFP antibody. The mean intensity of fluorescence per pixel was measured using ImageJ software and multiplied by the surface area of the growth cone to evaluate the binding of GFP-Sema3E per growth cone. Data were pooled from three independent experiments and mean was measured. The statistical significance of differences between the groups was evaluated using the Mann–Whitney test.

### FRET experiments

Dissociated E15.5 Pir neurons of wild-type CD1 or *Gipc1*^*−/−*^ mutant embryos were electroporated with the FRET biosensor Raichu R-ras and plated on glass-bottom dishes (MatTek). After 2 days in culture, neurons were imaged in a controlled atmosphere on the heated stage (37 °C) of an inverted spinning disk Eclipse TI microscope (Nikon) controlled by MetaMorph. Images for cyan fluorescent protein (CFP; excitation 445 nm, emission 495 nm) and yellow fluorescent protein (YFP; excitation 445 nm, emission 515 nm) were acquired with a 2 EMCCD camera (Photometrics) every 20 s from 6 min before to 12 min after the addition of Sema3E to the medium. YFP/CFP ratio images were generated with ImageJ software to represent the levels of FRET used for quantitative analyses. Mean ratios of randomly selected ROIs defined by individual vesicles were obtained for a period of 6 min (0–6 min, control) before Sema3E and were compared with the 6 min period (3–9 min) following Sema3E application. Changes of >10% of the mean control value were considered increases or decreases. Data are presented as percentage of vesicles showing increased, decreased or unchanged FRET levels. The statistical significance of differences between the groups was evaluated using the χ^2^ test.

### Immunohistochemistry

Brains were sectioned, at thickness of 80–100 μm, on a vibratome and immunohistochemistry on floating sections was performed following common procedures. Fluorescent-stained brain sections were imaged with an AxioImager Z1 Apotome controlled by Axiovision Imagery or with a confocal LSM 780 controlled by zen2010 software (Zeiss). The dorsoventral width of the AC was measured using ImageJ software at the level of the midline on all coronal sections through the body of the AC (2–3 sections per brain). The diameter and cross-sectional area of the AC were measured using ImageJ software on parasagittal sections taken at comparable mediolateral levels using the fornix as a landmark. The striatonigral tract was analysed on parasagittal sections in which the entire pathway could be seen from the globus pallidus to the substantia nigra (three sections per hemi-brain). The width of the tract was measured at mid-distance between the external border of the globus pallidus and the entopeduncular nucleus (visualized by DAPI staining), along an axis placed orthogonally to the orientation of the tract. For all analyses, 2–7 animals from at least 2 different litters were analysed for each genotype. The mean of measurements was calculated and statistical significance of differences between means was evaluated using the Mann–Whitney or the Kruskal–Wallis test.

For CTIP2/TRBR1 colocalization analyses, 2–3 animals from at least 2 different litters were analysed for each genotype. The percentage of co-labelled cells was calculated and statistical significance of differences between the groups was evaluated using the χ^2^ test.

Immunohistochemistry on whole-mount embryos with antibody against PECAM-1 was performed following standard procedures. Embryos were imaged with a Zeiss stereomicroscope Lumar. For each genotype, 5–15 animals from 3 different litters were analysed.

### *In situ* hybridization

*In situ* hybridization was performed on 100-μm-thick vibratome sections of E15.5 and E17.5 mouse brains following standard protocols. *Plxnd1* and *Sema3e* probes were generously provided by M. Tessier-Lavigne and C. Christensen^70^, respectively. The sequence 1,083 to 1,576 of the 3′ untranslated region of *Gipc1* cDNA (NM-018771.3) was cloned into pCR Topo Blunt II to obtain a *Gipc1* probe template. The vector was linearized by digestion with *Eco*RV, and antisense RNA probe was synthetized by Sp6 polymerase. The same vector was linearized by *Bam*HI and transcribed by T7 polymerase to obtain a sense RNA probe as a control.

### Axonal tracing

For axonal tracing, embryonic brains were fixed at least overnight in 4% PFA at 4 °C. Small crystals of DiI (1,1′-dioctadecyl 3,3,3′,3′-tetramethylindocarbo-cyanine perchlorate; Molecular Probes) were inserted into the cerebral cortex and allowed to diffuse at 37 °C for 2 weeks. Brains were cut into 100-μm-thick vibratome sections, and tracing specificity was systematically confirmed after diffusion on serial sections adjacent to the site of crystal insertion. Fluorescent-stained brain sections were imaged with an AxioImager Z1 Apotome controlled by Axiovision Imagery.

### Statistics

For each experiment, the normal distribution of the data was examined using a D'Agostino–Pearson omnibus test for sample sizes of 6 or higher. The estimate of variance was determined by the s.d. of each group that was similar between groups. Since data were nonparametric, Mann–Whitney test was used to compare two group means and Kruskal–Wallis test to compare differences between more than two groups. When data were distributed across categories, we used the χ^2^ test. All analyses were performed using the Prism6 software. Statistical significance was set at *P*<0.05. No statistical methods were used to predetermine sample sizes, but our sample sizes are similar to those generally employed in the field.

### Data availability

All relevant data are available from the authors.

## Additional information

**How to cite this article:** Burk, K. *et al*. Post-endocytic sorting of Plexin-D1 controls signal transduction and development of axonal and vascular circuits. *Nat. Commun.* **8,** 14508 doi: 10.1038/ncomms14508 (2017).

**Publisher's note:** Springer Nature remains neutral with regard to jurisdictional claims in published maps and institutional affiliations.

## Supplementary Material

Supplementary Information Supplementary Figures and Supplementary Reference.
