# Supplementary material for: Post-endocytic sorting of Plexin-D1 controls signal transduction and development of axonal and vascular circuits
Source: Nat Commun. 2017 Feb 22;8:14508. doi: 10.1038/ncomms14508 (PMC5322531; doi:10.1038/ncomms14508)
Supplement: Supplementary Information — Supplementary Figures and Supplementary Reference. [file ncomms14508-s1.pdf]

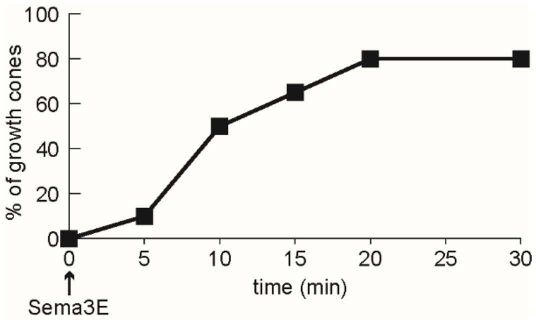

Supplementary Figure 1: Kinetic analysis of growth cone collapse.

Quantification of the percentage of growth cone collapse in culture of E15.5 Pir neurons imaged for 30 min after bath application of 10 nM Sema3E. n=21 growth cones

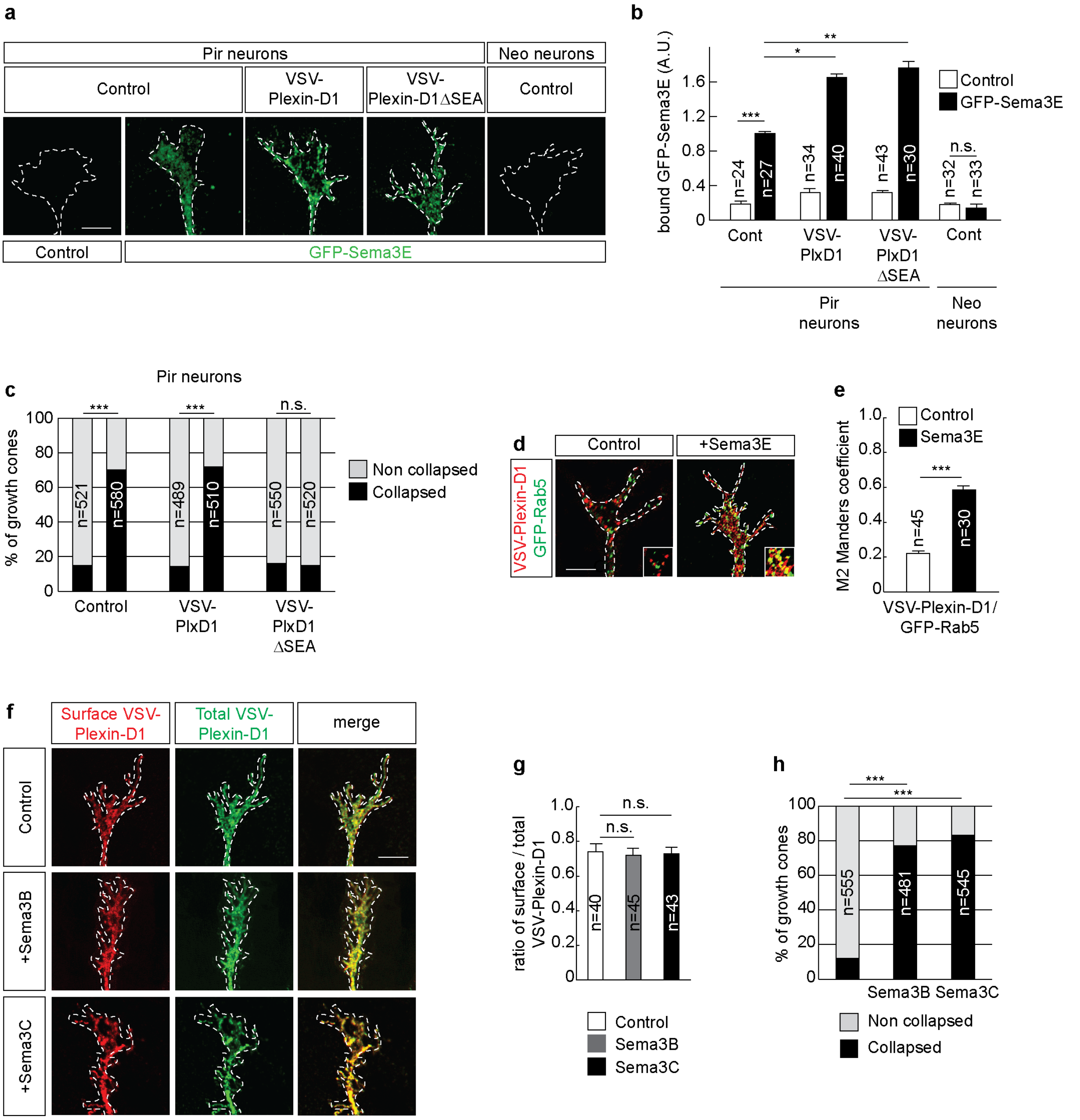

Supplementary Figure 2: The ligands Sema3B, Sema3C and Sema3E induce growth cone collapse of Pir neurons, but only Sema3E triggers endocytosis of the receptor Plexin-D1.

(a) Binding of GFP-tagged Sema3E to growth cones of E15.5 Pir or neocortical (Neo) neurons expressing or not VSV-Plexin-D1 and VSV-Plexin-D1ΔSEA.

(b) Quantification of GFP-Sema3E binding. Binding was 1.6 times and 1.7 times higher on Pir neurons expressing VSV-Plexin-D1 and VSV-Plexin-D1ΔSEA, respectively, compared to non-transfected neurons. GFP-Sema3E does not bind to Neo neurons, which do not

endogenously expressed Plexin-D1. n= number of growth cones analysed per condition in three independent experiments. Data are presented as mean ± SEM and values are indicated in arbitrary units (A.U.) of fluorescence, * $p < 0.05$, ** $p < 0.01$ by the Kruskal-Wallis test.

(c) Percentage of collapsed growth cones in cultures of E15.5 Pir neurons overexpressing or not VSV-Plexin-D1 and VSV-Plexin-D1ΔSEA and treated with Sema3E (20 min). Overexpression of Plexin-D1 does not cause hyperactivation of the Sema3E pathway. n= number of growth cones analyzed per condition in three independent experiments. Chi-square test, *** $p < 0.0001$.

(d) Growth cones of cultured E15.5 Pir neurons expressing GFP-Rab5 (green) and VSV-Plexin-D1 (red), with or without Sema3E treatment (10 min).

(e) Graph shows the Manders colocalization coefficient for the fraction of VSV-Plexin-D1 colocalized with GFP-Rab5. Sema3E increased colocalization of Plexin-D1 and Rab5. n= number of growth cones analyzed per condition in three independent experiments. Data are represented as mean ± SEM, *** $p < 0.0001$ by the Mann-Whitney test.

(f) Growth cones of E15.5 Pir neurons immunolabeled for surface (red) and total (green) VSV-Plexin-D1 after a 10 min treatment with Sema3B or Sema3C.

(g) Quantification of the cell surface/total VSV-Plexin-D1 ratio. Sema3B or Sema3C did not trigger Plexin-D1 endocytosis. n= number of growth cones analyzed per condition in three independent experiments. Data are represented as mean ± SEM, No statistical difference was found between conditions using the Kruskal-Wallis test.

(h) Percentage of collapsed growth cones of E15.5 Pir neurons with or without Sema3B or Sema3C treatment (20 min). Both ligands induce collapse. n= number of growth cones analyzed per condition in three independent experiments. Chi-square test, *** $p < 0.0001$.

Scale bars: 10 μm.

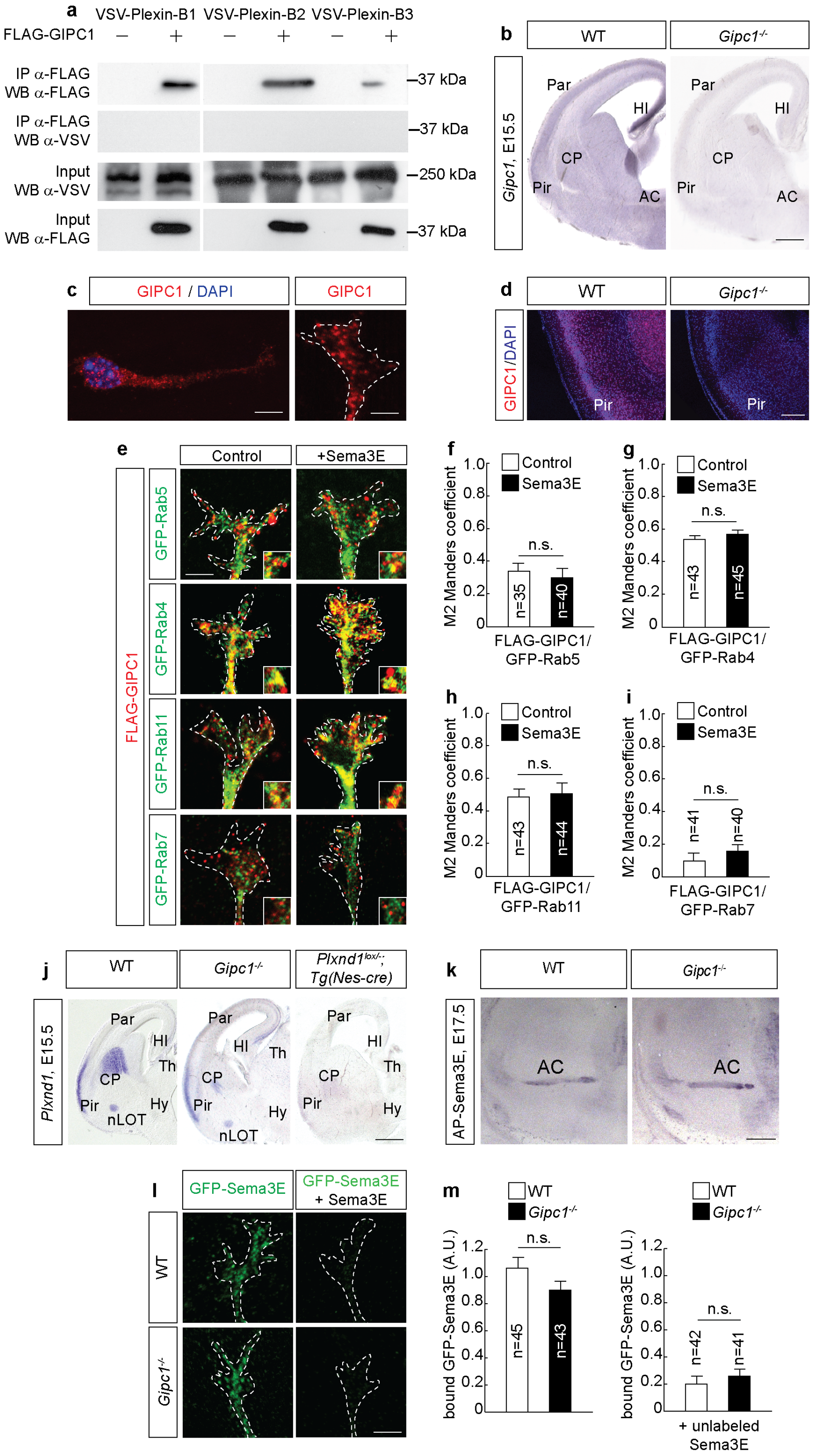

Supplementary Figure 3: Neuronal expression and binding properties of GIPC1.

(a) HEK293T cells were transfected with the indicated plasmids. Proteins were immunoprecipitated (IP) from cell lysates and immunoblotted (WB) using the indicated antibodies.

(b) Coronal sections of E15.5 wild-type (WT) or *Gipc1*$^{-/-}$ brains hybridized with an RNA probe for *Gipc1*.

(c) Cultured E15.5 Pir neurons immununostained with anti-GIPC1 antibody.

(d) Coronal sections through the Pir cortex of P6 wild-type (WT) or *Gipc1*$^{-/-}$ brains immunostained with anti-GIPC1 antibody.

(e) Growth cones of E15.5 Pir neurons expressing FLAG-GIPC1 (red) and different GFP-tagged Rab proteins (green) treated or not with Sema3E (10 min).

(f-i) Graphs showing the Manders colocalization coefficients for the fraction of FLAG-GIPC1 colocalized with GFP-Rab proteins. GIPC1 was detected on early and recycling endosomes and much less on late endosomes. This localization was independent of the presence of Sema3E. n= number of growth cones analyzed per condition in three independent experiments. Data are represented as mean ± SEM. No statistical difference was observed between conditions using the Mann-Whitney test.

(j) Coronal sections of E15.5 wild-type (WT), *Gipc1*$^{-/-}$ and *Plxnd1*$^{lox/-}$*;Tg(Nes-cre)* mouse brains were hybridized with RNA probe for *Plxnd1*. Signal can be seen in the Pir, CP, nLOT of the WT and *Gipc1-/-* brains, but is absent in the brain of *Plxnd1* mutants.

(k) Binding of AP-Sema3E to coronal brain sections from E17.5 WT and *Gipc1*$^{-/-}$ embryos. Labeling of the AC axon tract was seen in both genotypes.

(l) Binding of GFP-tagged Sema3E to growth cones of E15.5 WT and *Gipc1*$^{-/-}$ Pir neurons. Specificity of binding is shown by competition with unlabeled ligand.

(m) Quantification of GFP-Sema3E binding. Results indicate comparable binding on both types of neurons. n= number of growth cones analyzed per condition. Data are presented as mean ± SEM and values are indicated in arbitrary units (A.U.) of fluorescence. No statistical difference was found between genotypes by the Mann-Whitney test.

AC: anterior commissure, CP: caudate putamen, HI: hippocampus, Hy, hypothalamus ; nLOT, nucleus of the olfactory tract, Par: parietal cortex, Pir: piriform cortex, Th, thalamus. Scale bar: 400 µm (b, j), 300 µm (k), 200 µm (d), 50 µm (c *left panel*), 10 µm (c *right panel*, e, l).

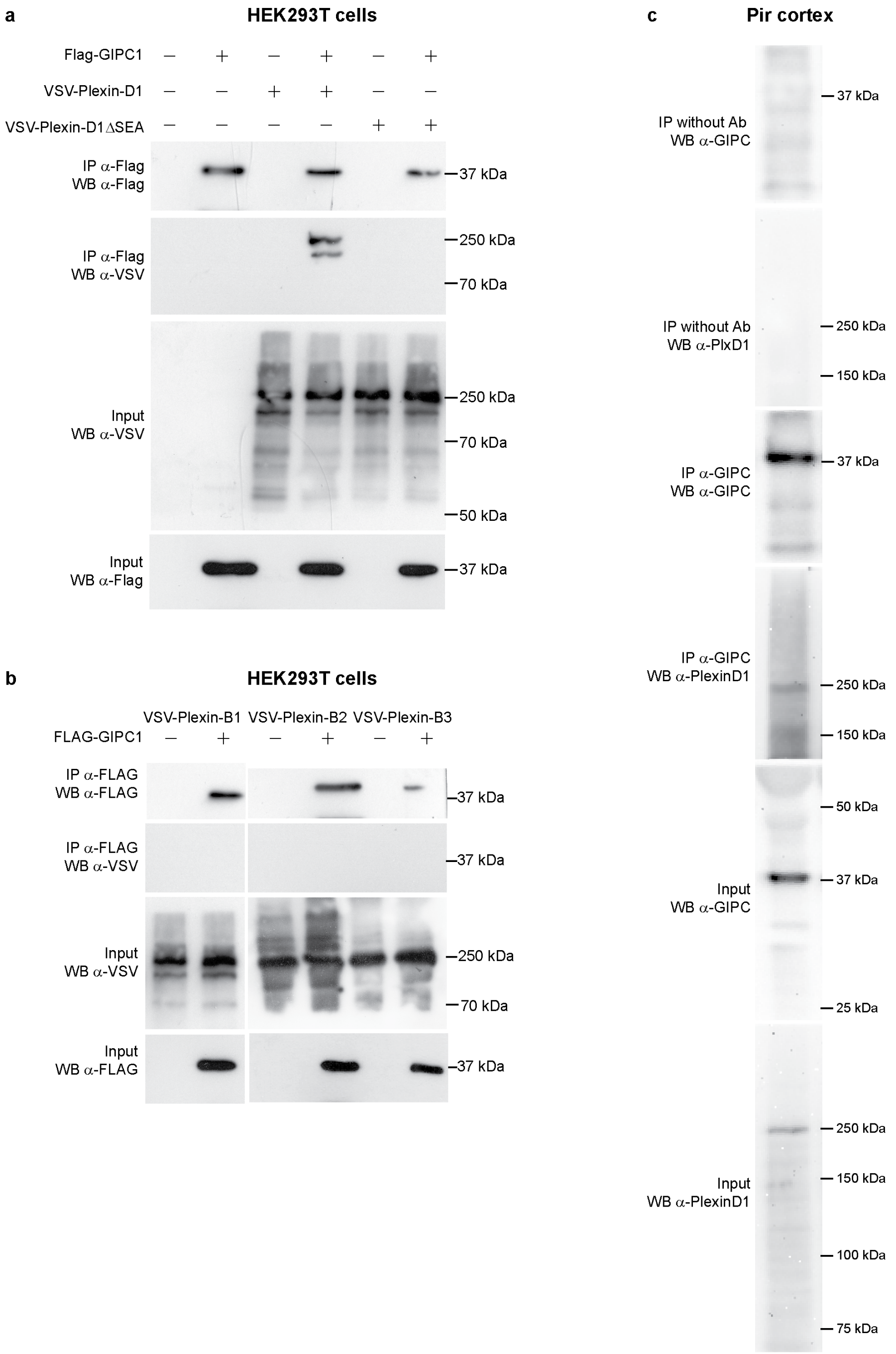

Supplementary Figure 4: Full blots of main Figure 4 and Supplementary Figure 3

(a) Full blot of main Figure 4a

(b) Full blot of Supplementary Figure 3a.

(c) Full blot of main Figure 4b.

For co-IP experiments, membranes were cut according to molecular weights to enable blotting for multiple antibodies and remove light and heavy chains of immunoglobulins.

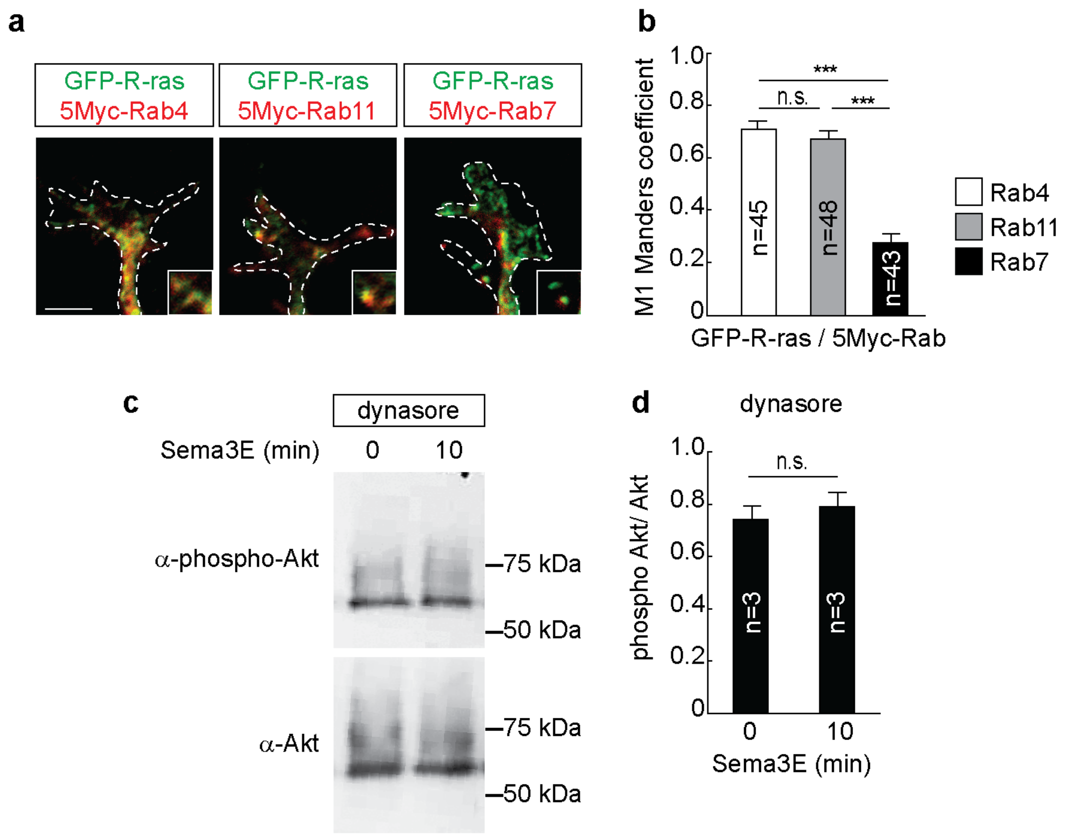

Supplementary Figure 5: Endosomal localization of R-ras and role of endocytosis in Sema3E-induced inhibition of Akt.

(a) Representative confocal images of growth cones of cultured E15.5 Pir neurons expressing GFP-R-ras (green) and the indicated 5Myc-tagged Rab proteins (red).

(b) Graphs showing the Manders colocalization coefficient for the fraction of GFP-R-ras colocalized with 5Myc-Rab4, 5Myc-Rab11 or 5Myc-Rab7. R-Ras colocalises preferentially with Rab4 and Rab11 and less with Rab7. n= number of growth cones analyzed per condition in three independent experiments. Data are represented as mean ± SEM. *** $p < 0.0001$ by the Kruskal-Wallis test.

(c) Phosphorylation of Akt in E15.5 Pir neurons stimulated or not with Sema3E (10 min) in the presence of dynasore. Blots shown are representative of three experiments.

(d) Densitometric quantification of phospho-Akt levels after normalization to total Akt in the experiments illustrated in panels (c). Sema3E-induced inhibition of Akt required endocytosis. n= number of experiments. Data are represented as mean ± SEM. No statistical difference was found between conditions using the Mann-Whitney test.

Scale bar: 10 µm

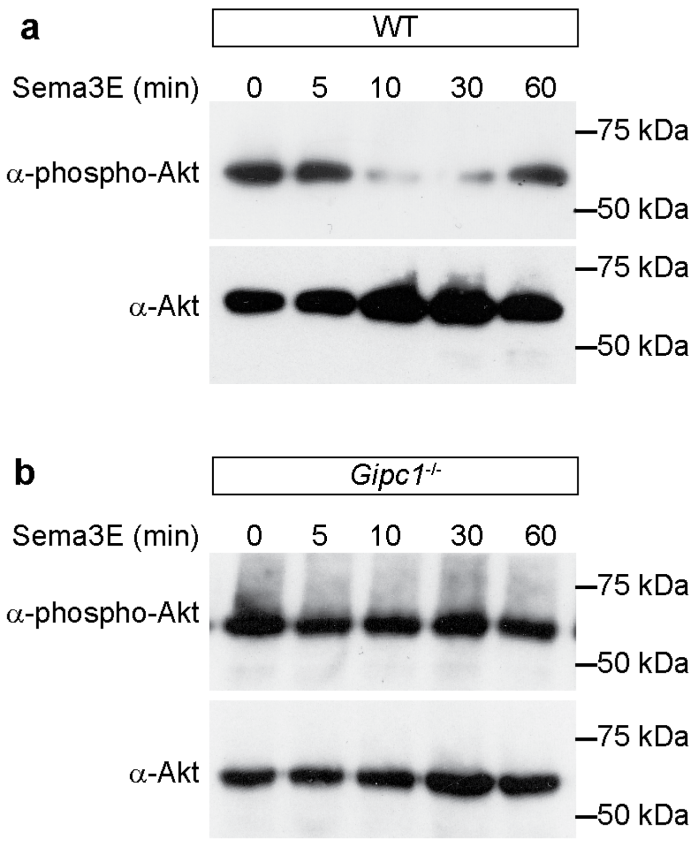

Supplementary Figure 6: Full blots of main Figure 7

(a) Full blot of main Figure 7i.

(b) Full blot of main Figure 7k.

After transfer nitrocellulose membranes were cut into strips to save antibody solutions.

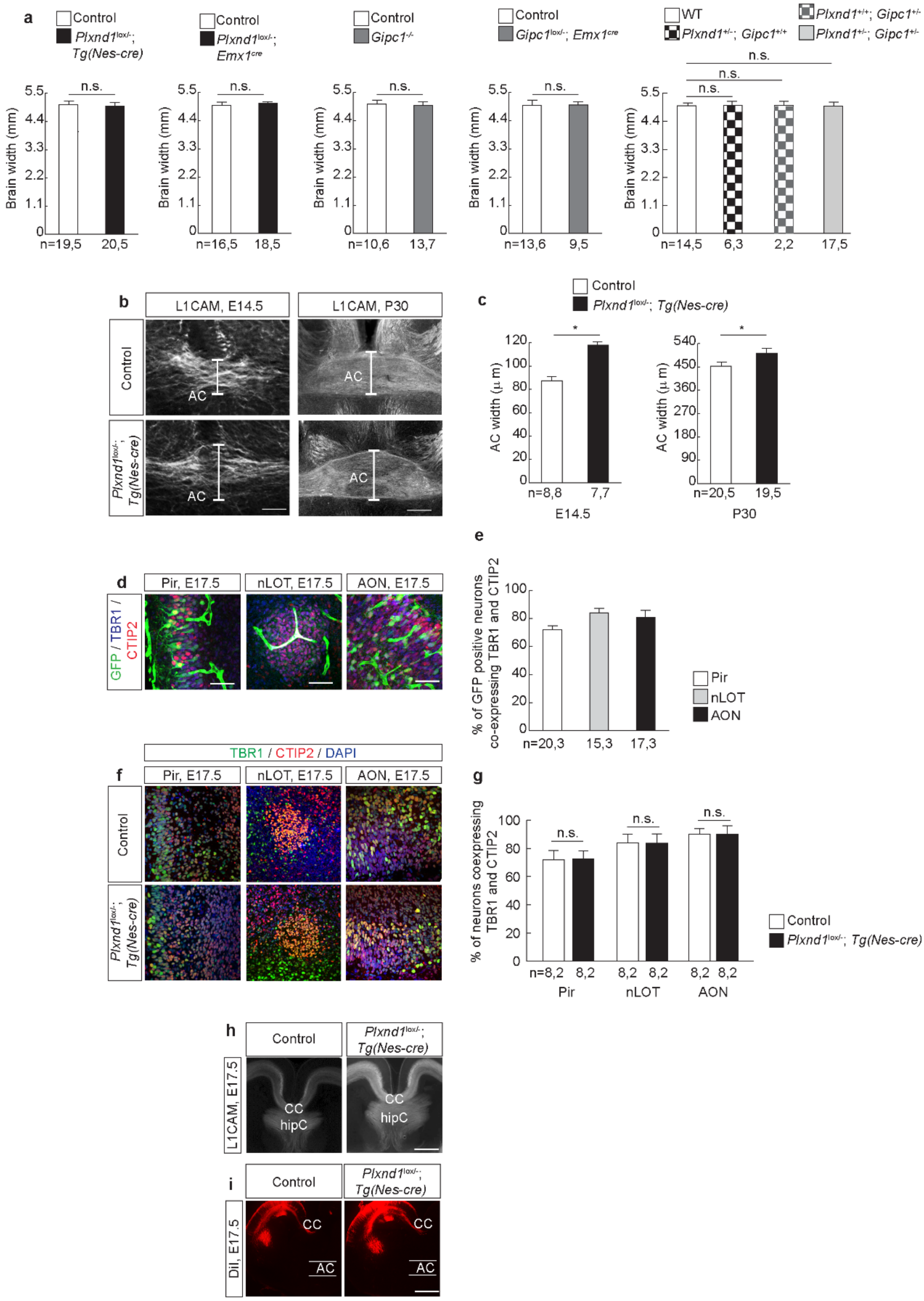
a
Control
Plxnd1lox/-; Tg(Nes-cre)
Plxnd1lox/-; Emx1cre
Gipc1-/-
Gipc1lox/-; Emx1cre
WT
Plxnd1+/-; Gipc1+/+
Plxnd1+/+; Gipc1+/-
Plxnd1+/-; Gipc1+/-
n.s.
Brain width (mm)
n=19,5 20,5
n=16,5 18,5
n=10,6 13,7
n=13,6 9,5
n=14,5 6,3 2,2 17,5
b
L1CAM, E14.5
L1CAM, P30
AC
c
AC width (µ m)
n=8,8 7,7
E14.5
n=20,5 19,5
P30
d
Pir, E17.5
nLOT, E17.5
AON, E17.5
GFP / TBR1 / CTIP2
e
% of GFP positive neurons co-expressing TBR1 and CTIP2
n=20,3 15,3 17,3
Pir
nLOT
AON
f
TBR1 / CTIP2 / DAPI
g
% of neurons coexpressing TBR1 and CTIP2
n=8,2 8,2 8,2 8,2 8,2 8,2
h
L1CAM, E17.5
CC
hipC
i
DiI, E17.5

Supplementary Figure 7: Analysis of the role of Plexin-D1 in the development of the AC.

(a) Brain width at the level of the AC in E17.5 mouse embryos of the indicated genotypes. Data are represented as mean ± SEM, n=x,y where x indicates the number of slices and y the number of mice analysed for each genotype. No statistical difference was found between conditions using the Mann-Whitney test and the Kruskal-Wallis test.

(b) L1CAM-stained AC in coronal sections of E14.5 and P30 brains from control and *Plxnd1*$^{lox/-}$*;Tg(Nes-cre)* mice.

(c) Quantification of AC width. Mice lacking Plexin-D1 developed an enlarged AC. Data are shown as mean ± SEM, n=x,y where x indicates the number of slices and y the number of mice analysed for each genotype. * $p < 0.05$ by the Mann-Whitney test.

(d) Triple immunochemistry for GFP, T-box brain 1 (TBR1) and COUP TF1- interacting protein 2 (CTIP2) on coronal brain sections of E17.5 mouse embryos expressing GFP under the control of the *Plxnd1* promoter (*Tg(Plxnd1-EGFP)HF78Gsat/Mmcd* mice).

(e) Percentage of GFP-positive neurons coexpressing TBR1 and CTIP2. Data are shown as mean ± SEM, n=x,y where x indicates the number of slices and y the number of mice analysed.

(f) Double immunochemistry for TBR1 and CTIP2 on coronal sections of E17.5 control or *Plxnd1*$^{lox/-}$*;Tg(Nes-cre)* mutant brains.

(g) Percentage of neurons coexpressing TBR1 and CTIP2. *Plxnd1* deletion did not affect the generation and specification of neurons. Data are shown as mean ± SEM, n=x,y where x indicates the number of slices and y the number of mice analysed for each genotype. No statistical difference was found between genotypes using the chi-square test.

(h, i) Anti-L1CAM staining of coronal sections through the CC and hipC (h) and DiI staining of CC projections (i) in E17.5 brains from control or *Plxnd1*$^{lox/-}$*;Tg(Nes-cre)* mutants. No sign of CC dysgenesis or misrouting of neocortical axons towards the AC was found in *Plxnd1*$^{lox/-}$*;Tg(Nes-cre)* mutants.

AC, anterior commissure; AON, anterior olfactory nucleus; CC, corpus callosum; hipC, hippocampal commissure, nLOT, nucleus of the olfactory tract; Pir, piriform cortex. Scale bars: 30 μm (d), 50 μm (f), 100 μm (b: P30), 200 μm (b: E14.5), 300 μm (i), 400 μm (h).

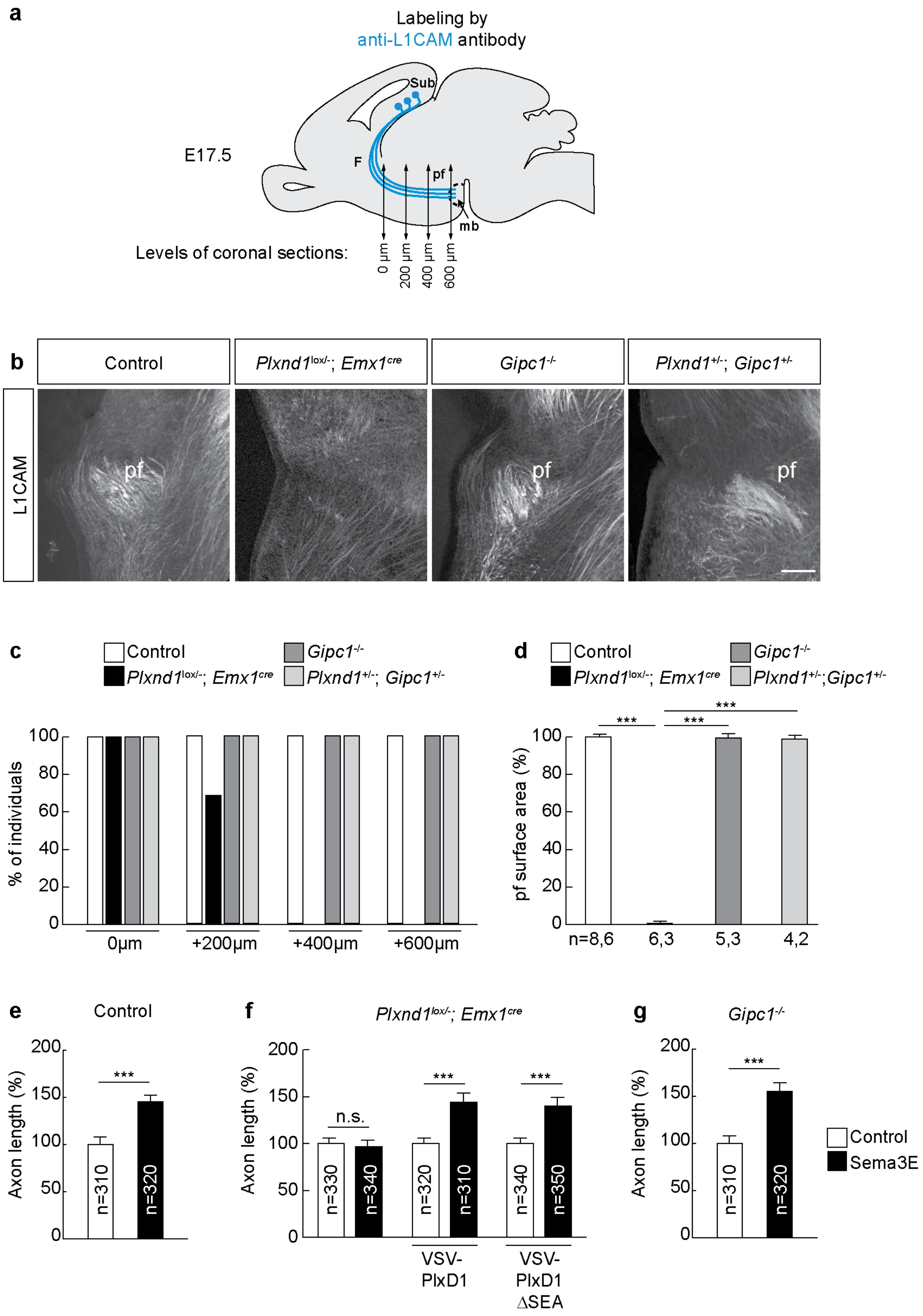

Supplementary Figure 8: *Gipc1* is dispensable for the development of the subiculo-mammillary axon tract.

(a) Schematic representation of the levels of coronal sections from the fornix (0 µm) to the caudal hypothalamus (+600 µm) used for analysis of the postcommissural fornix (pf) in E17.5

mouse brains.

(b) L1CAM immunostaining of the pf on coronal sections through the caudal hypothalamus (+600 μm) of E17.5 mouse brains from control, *Plxnd1*$^{lox/-}$*;Emx1*^{cre}, *Gipc1*$^{-/-}$ and double-heterozygous *Plxnd1*$^{+/-}$*;Gipc1*$^{+/-}$ mutants.

(c) Quantification of the percentage of coronal sections with a visible pf in E17.5 brains from control mice (n=10 mice), conditional *Plxnd1*$^{lox/-}$*;Emx1*^{cre} mutants (n=5 mice), null *Gipc1*$^{-/-}$ (n=4 mice) and double-heterozygous *Plxnd1*$^{+/-}$*, Gipc1*$^{+/-}$ (n=4 mice) mutants. Caudal extension of the pf required functional Plexin-D1, but not GIPC1.

(d) Quantification of the cross-sectional surface area of the pf in E17.5 brains from control mice, conditional *Plxnd1*$^{lox/-}$*;Emx1*^{cre} mutants, null *Gipc1*$^{-/-}$ and double-heterozygous *Plxnd1*$^{+/-}$*, Gipc1*$^{+/-}$ mutants. Mice lacking GIPC1 developed a normal subiculo-mammilary tract. Data are shown as mean ± SEM, n=x,y where x indicates the number of slices and y the number of mice analysed for each genotype. *** $p < 0.0001$, by the Kruskal-Wallis test.

(e-g) Quantification of axon length in cultures of subicular neurons from E17.5 control, *Plxnd1*$^{lox/-}$*;Emx1*^{cre} or *Gipc1*$^{-/-}$ mouse embryos, kept for 2 days in the absence or presence of Sema3E. Interaction between GIPC1 and the SEA motif in Plexin-D1 was not required to mediate the growth-promoting effect of Sema3E. Data are presented as mean axonal length ± SEM and are normalized to the values obtained in unstimulated conditions. n= number of axons measured per condition in three independent experiments. ***$p < 0.0001$, by the Mann-Whitney test.

F, fornix; pf, postcommissural fornix, Sub, subiculum; mb, mamillary bodies.

Scale bar: 110 μm.

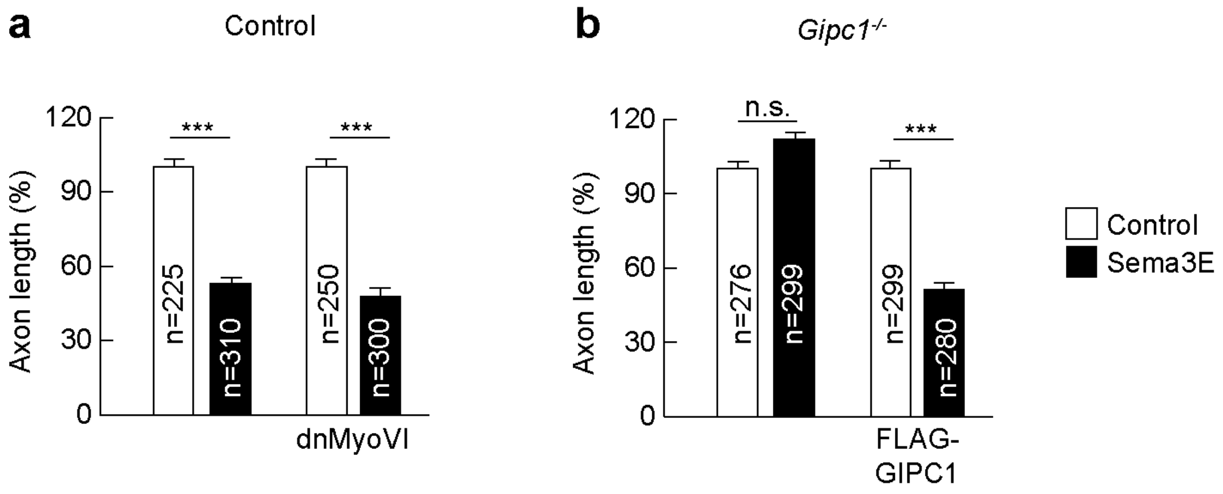

Supplementary Figure 9: Myosin VI is dispensable for the growth inhibitory effect of Sema3E

(a) Quantification of axon length in cultures of E15.5 Pir neurons transfected or not with a dominant negative Myosine VI (dnMyoVI) construct [1] and kept for 3 days in the presence or absence of Sema3E (5nM). In control condition, Sema3E inhibits axonal length by 50%. MyosinVI is not required for this inhibitory growth response. Data are presented as mean axonal length ± SEM and are normalized to the values obtained in unstimulated conditions. n= number of axons measured in three independent experiments.

***p < 0.0001, by the Mann-Whitney test.

(b) Quantification of axon length in cultures of E15.5 Pir neurons of *Gipc1*$^{-/-}$ mutants kept for 3 days in the presence or absence of Sema3E (5nM). Mutant axons lack sensitivity to inhibition by Sema3E. Expressing a recombinant FLAG-GIPC1 rescues growth inhibition. Data are presented as mean axonal length ± SEM and are normalized to the values obtained in unstimulated conditions. n= number of axons measured in three independent experiments.

***p < 0.0001, by the Mann-Whitney test.

Supplementary Reference

1. Lewis, T.L., Jr., Mao, T. & Arnold, D.B. A role for myosin VI in the localization of axonal proteins. *PLoS biology* **9**, e1001021 (2011).
